# A dataset of trophic state index for nation-scale lakes in China from 40-year Landsat observations

**DOI:** 10.1038/s41597-024-03506-7

**Published:** 2024-06-21

**Authors:** Minqi Hu, Ronghua Ma, Kun Xue, Zhigang Cao, Xi Chen, Junfeng Xiong, Jinduo Xu, Zehui Huang, Zhengyang Yu

**Affiliations:** 1 Key Laboratory of Watershed Geographic Sciences, Nanjing Institute of Geography and Limnology, Chinese Academy of Sciences, Nanjing, 210008 China; 2 University of Chinese Academy of Sciences, Nanjing, Nanjing, 211135 China; 3 School of Geography and Planning, Sun Yat-sen University, Guangzhou, 510275 China

**Keywords:** Limnology, Environmental impact

## Abstract

Trophic state index (TSI) serves as a key indicator for quantifying and understanding the lake eutrophication, which has not been fully explored for long-term water quality monitoring, especially for small and medium inland waters. Landsat satellites offer an effective complement to facilitate the temporal and spatial monitoring of multi-scale lakes. Landsat surface reflectance products were utilized to retrieve the annual average TSI for 2693 lakes over 1 km^2^ in China from 1984 to 2023. Our method first distinguishes lake types by pixels with a decision tree and then derives relationships between trophic state and algal biomass index. Validation with public reports and existing datasets confirmed the good consistency and reliability. The dataset provides reliable annual TSI results and credible trends for lakes under different area scales, which can serve as a reference for further research and provide convenience for lake sustainable management.

## Background & Summary

Trophic state index (TSI) is crucial and meaningful to lake ecosystems as a representative indicator of eutrophication^1,2^. Lake eutrophication can be strongly affected by constant sediment re-suspension and release of historically accumulated nutrients, which further exerts a profound impact on underwater light and primary production, and changes the structure and function of the lake ecosystem with the bottom-up effect^3,4^. Eutrophication causes the degradation of lake ecosystems, induces severe influences on aquatic plants, fish, and oxygen-sensitive invertebrates and threatens the drinking water supply^5,6^.

As a crucial variable in regional research, the variation of TSI influenced by global climate change and human activities had been widely explored, including impacts on the climate warming^7,8^, precipitation^9,10^, wind speed^11,12^, atmospheric sedimentation^13^, population^14,15^, nutrient application^16,17^, and hydrodynamics^18^. Continuous monitoring of worldwide lakes has reported eutrophication associated with global warming and rising population, leading to the propagation and outbreak of harmful algae blooms^19,20^, indicating the necessity of long-term observation of the lake eutrophication.

Although water quality parameters from field experiments provide a good source of TSI data for eutrophication observations, dispersed samples and significant cost render a challenge for continuous monitoring with long time series^2,21,22^. The process of transportation and sample pre-processing may lead to delayed measurements. In addition, due to multi-year climatic oscillations, even long-term field experiments miss potential phases of these climatic cycles that may affect the temporal patterns of trophic states, causing both spatial and temporal observation constraints^23^.

Remote sensing with frequent and extensive data acquisition, providing an essential approach for regional and global studies and effectively compensates for the labour-intensive and low-frequency problems of monitoring with manual sampling^24,25^. Traditional ocean colour sensors have been widely used to retrieve water parameters as well as TSI in several studies^26–29^. However, the moderate spatial resolution (250 m–1 km) limited the application to small and medium lakes^30^, and affects historical backtracking before 2000. Landsat satellites, with the considerable radiometric resolution (8–12 bit) and high spatial resolution (30–120 m), have been used efficiently to retrieve biological^31–33^ and physical^34,35^ parameters for coastal and inland water with more spatial details since the 1980s.

National-scale of lake trophic monitoring has always been challenged by different geographic contexts, as the dissolved and suspended solids of lakes are highly diverse among different regions of the Earth which may lead to different regression coefficients^16,24^. In China, Zhang *et al*.^36^ presented a mapping of trophic evaluation for national lakes over 10 km^2^ in 2020. Currently, the long-term remote sensing of the trophic state assessment for national-scale lakes are not present in China, particularly for small lakes (~1 km^2^), which are more densely distributed in China and provide similarly valuable ecosystem functions as large lakes^37^.

**Fig. 1:**
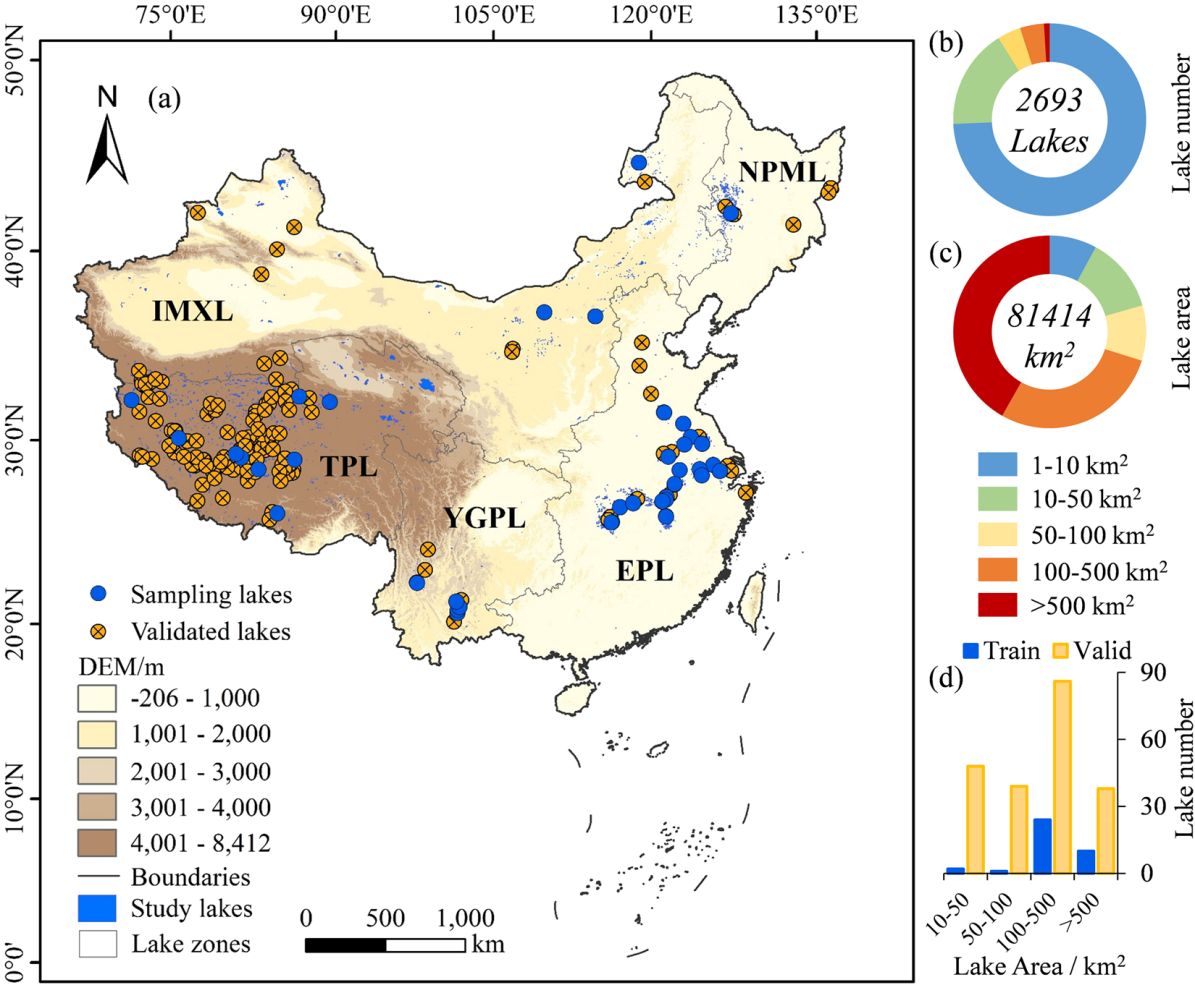
(**a**) Locations of studied lakes and lake zones. Proportions of (**b**) lake number and (**c**) average lake area under different lake scales were counted, including 1–10 km^2^, 10–50 km^2^, 50–100 km^2^, 100–500 km^2^ and above 500 km^2^. Sampling lakes and validated lakes were counted in (**d**) bar chart.

This study intends to overcome the gap by exploiting Landsat’s capabilities to provide a thorough understanding of the eutrophication trend of lakes in China. Therefore, the China Lake Trophic State Index (CNLTSI) dataset for 2693 lakes over 1 km^2^ in China (Fig. 1) was created based on Landsat products, which provided the annual average records and spatial distributions of TSI for each lake from 1984 to 2023. The purpose of this study is to (1) create the latest CNLTSI dataset in China using available 40-year Landsat images, (2) provide the temporal and spatial heterogeneity of CNLTSI for nationwide lakes on a 30-meter resolution, and (3) distribute and share CNLTSI data to citizens, scholars for further research, and facilitate decision-making processes for government.

## Methods

The CNLTSI dataset is generated for 2693 natural lakes over 1 km^2^ in China. These lakes are distributed in the five lake zones according to the lake origin, topography, geomorphology and climatic characteristics (Fig. 1a): (1) the Eastern Plain Lake Zone (EPL, N = 629), (2) the Northeast Plain and Mountain Lake zone (NPML, N = 417), (3) the Inner Mongolia-Xinjiang Lake Zone (IMXL, N = 522), (4) the Yunnan-Guizhou Plateau Lake Zone (YGPL, N = 64), and (5) the Tibetan Plateau Lake Zone (TPL, N = 1061)^38^. This dataset includes lakes with surface areas over 1 km^2^, among which lakes ranging from 1–10 km^2^ account for 74.30% of the total number (Fig. 1b). Only 0.97% of lakes larger than 500 km^2^, and the area accounts for 42% of the total lake area in the past four decades (Fig. 1c).

**Fig. 2:**
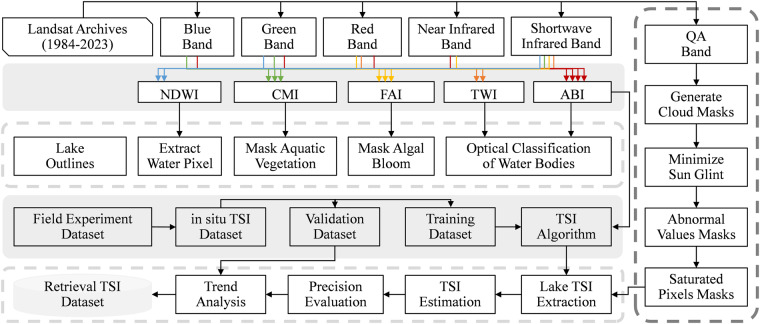
Workflow and methods for generating TSI dataset from Landsat archives.

Figure 2 depicts the workflow for the TSI inversion and dataset production. The CNLTSI dataset was created by applying TSI retrieval algorithms to visible light, NIR, and SWIR bands, as well as metadata from Landsat data. The quality assurance (QA) band delineating the surface, atmospheric, and sensor conditions included in the Landsat data was used to mask clouds and other obstacles^25^. Cloud pixels with high (67% – 1 00%) and medium (34% – 66%) coverage values were masked, whereas low values (0% – 33%) were saved as cloud-free^23^. Cloud masks were further applied to reduce cloud-distorted pixels. The initial position of each lake was defined by the lake outlines dataset^39^, and the water pixel were further extracted. Details of the procedures and methods are provided in the following sections.

### Landsat series imagery

All available surface reflectance (SR) of Landsat imagery (Level 2, Collection 1, Tier 1), including Landsat-5 TM, Landsat-7 ETM+ and Landsat-8 OLI, were archived on the Google Earth Engine (GEE) platform to monitor the spatiotemporal dynamics of lake trophic states. Surface reflectance of TM and ETM+ were recalibrated to comparable levels of OLI using the calibration coefficients to generate a unified dataset to minimize spectral differences between multiple Landsat instruments^40^. The Land Surface Reflectance Code (LaSRC) and Landsat Ecosystem Disturbance Adaptive Processing System (LEDAPS) algorithms were applied to minimize the effects of absorption and scattering from atmosphere^41^. The solar zenith and azimuth angles of each image were used with the Shuttle Radar Topography Mission digital elevation model to simulate and remove terrain shadows^42^. Landsat tiles were downloaded from the United States Geological Survey (USGS) official website. The studied lakes in China are covered by 296 Landsat tile scenes, each with 5000 × 5000 30 m pixels with overpass times between 18:00 and 20:00 UTC. Landsat instruments have a 16-day revisit period and orbit at 705 km altitude.

### Lake outlines and properties data

Location, area, and outline of natural lakes were obtained from Lake-Watershed Science SubCenter, National Earth System Science Data Center, National Science & Technology Infrastructure of China (http://gre.geodata.cn). This database provides a complete and reliable spatial distribution of lakes nationwide with basic properties^39,43^. Lake outlines were interpreted based on China Brazil Earth Resources Satellite (CBERS) and Landsat-5 TM, combined with topographic maps and China Lake Chronicles^43^. In all, 2693 individual lake vector polygons over 1 km^2^ are contained, and the existence of these lakes were checked during the study period. The elevation of each lake was obtained from the NASA Shuttle Radar Topography Mission (SRTM) Digital Elevation 30 m. The SRTM V3 product (SRTM Plus) created digital elevation models under a near-global scale^42^ and was archived from GEE at a resolution of 1 arc-second (approximately 30 m). The average elevation was calculated as the elevation attribute for each lake.

### Training dataset

A total of 1114 water samples from 37 lakes (Fig. 1a,Table S1) were collected between 2013 and 2021 concurrent with Landsat overpass. Two of the sampled lakes have an area between 10–50 km^2^, one lake has an area between 50–100 km^2^, 24 lakes have an area between 100–500 km^2^, and 10 lakes have an area greater than 500 km^2^ (Fig. 1d). These lakes are distributed across various climate zones and lake zones, each with its own geographic significance. Due to accessibility constraints, fewer samples were collected in the western plateau of China (Fig. 1a). The sampled lakes range in area from 35.41 to 3192 km², with measured TSI varying from 20 to 90, encompassing a variety of water quality parameters and optical types (Table S1). All samples were collected in the epilimnion of the photic zone. A number of 649 water samples concurrent with satellite transit were used for model training and the remaining were used for independent validation. The water quality of sampled lakes in China were described in Supplementary Text 1. Secchi Disk Depth (SDD, m) was measured using a circular white-and-black disk with 30 cm diameter and the distance from water surface to unobservable disk was recorded as an SDD value^44^. Water samples were then transported to the laboratory and filtered with 47-mm Whatman GF/F glass fiber filters. The chlorophyll-a concentration (Chla, μg·L^−1^) was obtained under 90% acetone and determined spectrophotometrically using a Shimadzu UV2600 spectrophotometer^45^. The concentration of TP (mg·L^−1^) were determined through a spectrophotometric analysis after the potassium persulfate digestion^46^.

### Validation dataset

The accuracy of the generated TSI dataset was verified by numerical comparison and trophic level pairing. The external validation dataset for numerical comparison comes from lake sampling concurrent with satellite transit, including: (1) field experiments (446 pairs in 112 lakes, Table S2), (2) the investigation report on lakes in China (73 pairs in 73 lakes, Table S3, http://www.lakesci.csdb.cn), (3) water quality investigation of lakes on the Tibetan Plateau from 2009 to 2019^47^ and (4) monthly experiments from fixed stations in Lake Taihu by Taihu Laboratory for Lake Ecosystem Research. The validation dataset for level pairing consists of (1) 2112 trophic level records for 125 lakes calculated from remote-sensed Forel-Ule index during 2000–2018^48^ (Table S4, 10.6084/m9.figshare.13014299^49^), (2) 265 trophic level records for 29 lakes reported by the China Environmental Bulletin (Table S5, http://www.cnemc.cn/jcbg/zghjzkgb), and (3) 297 records for 52 lakes collected from published papers (Table S6). The time scale of each validation dataset keeps consistent in the accuracy evaluation.

### Assessment of lake trophic state

The trophic state index (TSI) was proposed by Carlson (1977) to incorporate most lakes in a scale of 0 to 100 and represent the variation in algal biomass. The index can be calculated from SDD, Chla and TP^1^. However, the complex optical characteristics of shallow lakes have been proven to affect the consistency of TSI calculated from different parameters, as SDD can be influenced by the abundance of organic and inorganic particulate and dissolved matter^50,51^. Chla concentration is an indicator for the bioproductivity of a lake based on the efficiency of photosynthesis^52^. In this study, we compared the TSI calculated from Chla and other indicators (Supplementary Text 2). TSI(Chla) showed the best correlation (R^2^ = 0.87, p < 0.01, N = 143) with multi parameters, illustrating the reliability of Chla as a benchmark for assessing eutrophication. Therefore, a numerical indicator that was calculated from Chla to assess the lake trophic state based on the Eq. (1):

1 $${\rm{TSI}}\,({\rm{Chla}})\,=\,{\rm{10\times }}\,({\rm{2}}{\rm{.5\; +\; 1}}{\rm{.086}}\,\mathrm{lnChla})\,,$$

In the TSI range of 0–100, 0–30 represents oligotrophic, 30–50 represents mesotrophic, 50–60 represents light eutrophic, 60–70 represents moderate eutrophic, and 70–100 represents hyper eutrophic^1^.

### Water extraction and classification

The initial boundary of each lake was extracted by lake outlines. The normalized difference water index (NDWI, Eq. 2) was applied to distinguish water pixels based on Landsat green band (555 nm) and near-infrared band (859 nm)^53^, and the threshold of each lake was defined by the bimodal threshold^54^.

2 $${\rm{NDWI}}={\rm{G}}-{\rm{NIR,}}$$

A three-pixel (~ 100 m) buffer was created to reduce the influence of water pixels mixing with land boundary pixels. NDWI was further used to distinguish cyanobacterial scums and aquatic macrophytes^55^, and the floating algal index (FAI, Eq. 3) was combined to improve the extraction of aquatic vegetation pixels, which has been confirmed to be effective for shallow lakes in China^56^:

3 $${\rm{FAI}}={\rm{NIR}}-{\rm{R}}-({\rm{SWIR}}-{\rm{R}})\times ({\lambda }_{{\rm{NIR}}}-{\lambda }_{{\rm{R}}})/({\lambda }_{{\rm{SWIR}}}-{{\rm{\lambda }}}_{{\rm{R}}}),$$

The unified threshold is used for each satellite image: high suspended water pixels are removed using a threshold FAI < −0.01, and thick floating algae pixels are removed using a FAI > 0.02^57^. The gradient method is used to determine the FAI threshold of aquatic vegetation extraction is −0.004 based on the residual pixels^16^.

Water pixels were divided into three types based on the spectral characteristics of lakes under different trophic levels (Supplementary Text 3): algae dominated (Type 1), turbid (Type 2), and clear pixels (Type 3). The optical signal can be affected in highly turbid lakes, leading to the recognition of high turbidity areas as eutrophic waters. Therefore, a turbid water index (TWI) was proposed to avoid the misclassification^58^ (Eq. 4):

4 $${\rm{TWI}}={\rm{R}}-{\rm{SWIR,}}$$

where G, R, NIR and SWIR correspond to the surface reflectance of OLI at 563 nm, 665 nm, 865 nm and 1609 nm, and the surface reflectance of TM and ETM + at 560 nm, 660 nm, 835 nm and 1650 nm, respectively, λ is the corresponding wavelength. The TWI threshold for turbid water (Type 2) is determined to be 0.076 based on the 95% confidence interval, and pixels with TWI < 0.076 are labelled as Type 1 water pixels dominated by algae^16^.

**Fig. 3:**
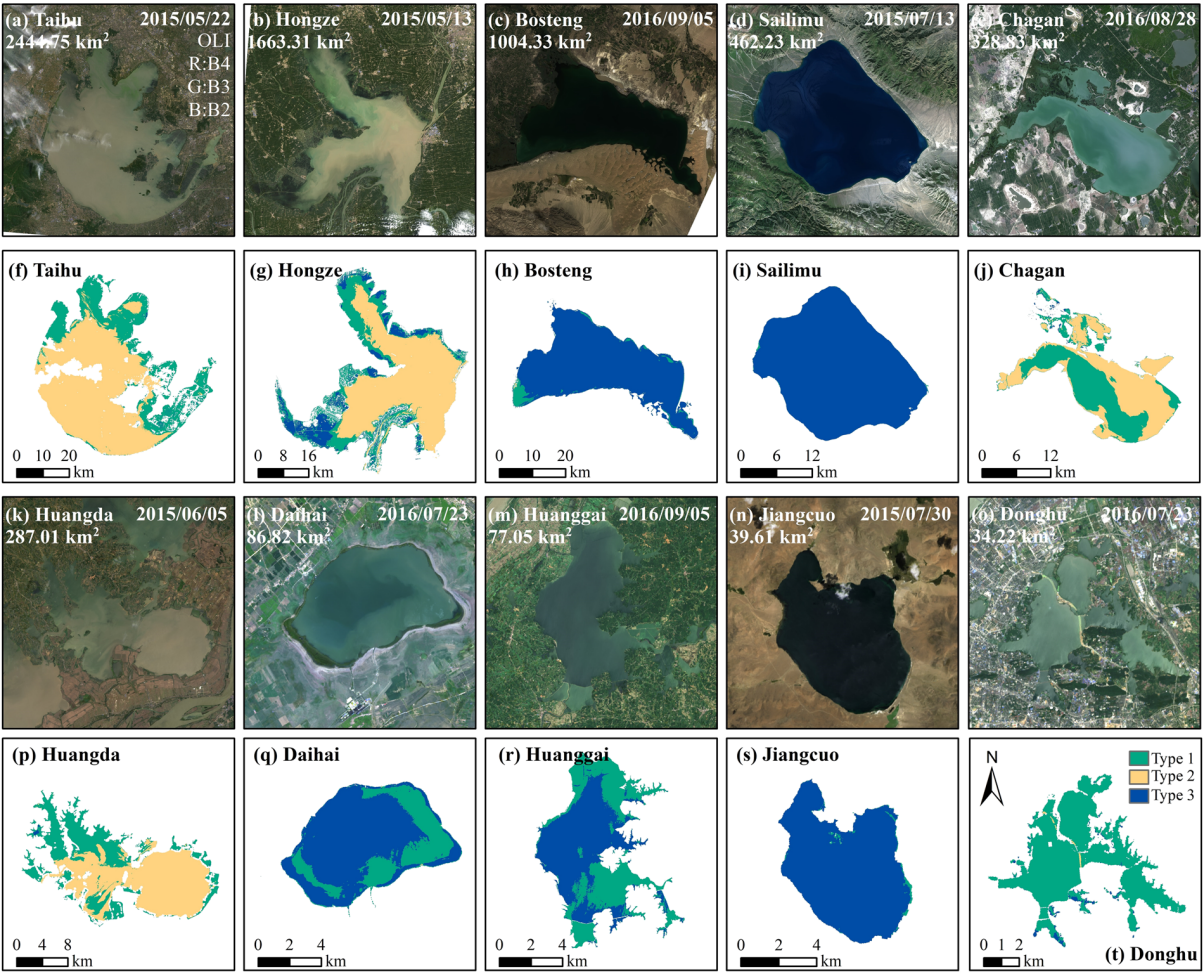
Landsat-8 OLI images of lakes with different surface area displayed by Band 4, 3 and 2 channels and corresponding water pixel classifications.

Almost no algae or suspended solids exist in relatively clear waters, especially in some deep lakes in the TPL^47,59^. The spectral reflectance was low due to the absorption of water bodies, especially after red band, and sometimes showed higher reflectance in the blue band than green band, which is similar to the ocean spectrum^60^. We further extracted absolute values of green band reflectance from surveyed oligotrophic lakes to pick out clear water pixels. Based on the near normal distribution with a mean (μ) and standard deviation (σ) of 0.065 and 0.021, the threshold for clear water pixel was 0.11 (μ + 2σ) with 95% confidence interval. Therefore, the criteria for determining a clear water pixel (Type 3) was determined under any of the following conditions: (1) the reflectance of green band is lower than 0.11; (2) the reflectance of blue band is greater than that of the green band. The classification mappings of lake water bodies under different area scales are shown in Fig. 3.

### Lake trophic state modelling

**Fig. 4:**
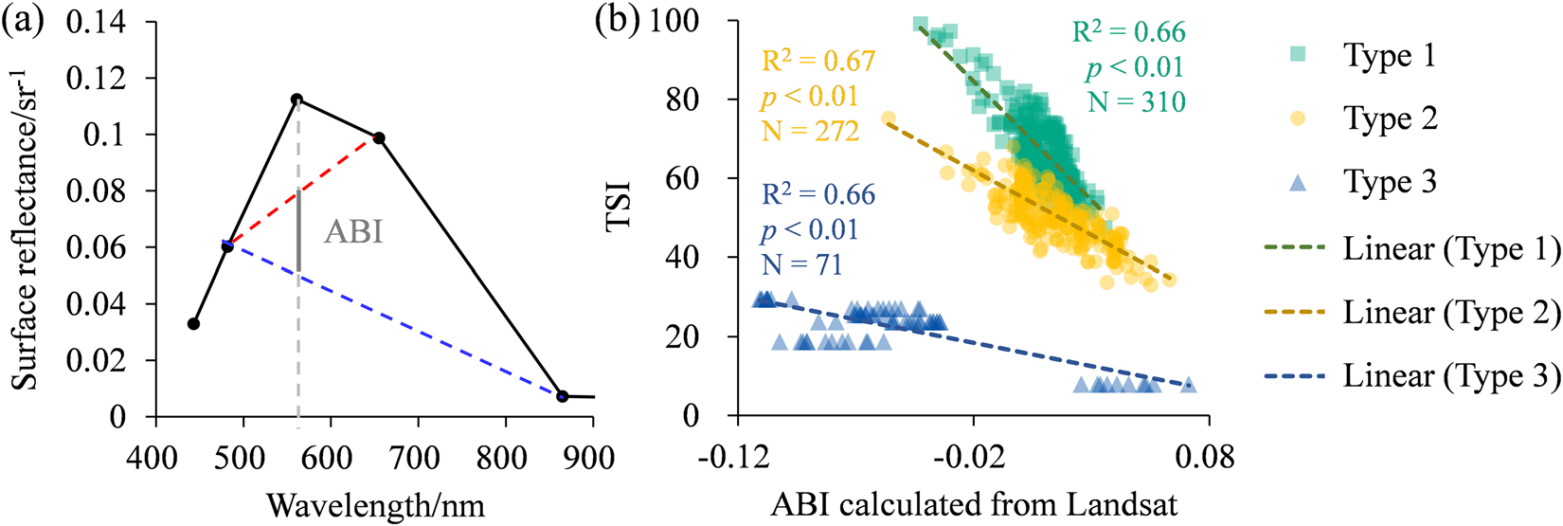
(**a**) Baseline structure of ABI, and (**b**) fitting model of ABI-derived TSI.

Algal biomass is a key descriptor in the construction concept of the TSI, and each major division of TSI represents a doubling of algal biomass, which became the essence of eutrophication assessment^61^. The algal biomass index (ABI) was proposed to realize the remote sensing estimation of algal biomass in shallow lakes, and was immune to sun glint and thickness of aerosols^62^. Therefore, the study attempted to detect the trophic state of lake through ABI directly. ABI is defined as the difference in remote-sensing reflectance at green band normalized against two baselines with one formed linearly between the near-infrared and blue band, and another formed linearly between red and blue band^16^ (Fig. 4a, Eq. 5):

5 $${\rm{ABI}}=({\rm{R}}-{\rm{B}})\times \frac{{\lambda }_{{\rm{G}}}-{\lambda }_{{\rm{B}}}}{{\lambda }_{{\rm{R}}}-{\lambda }_{{\rm{B}}}}-({\rm{NIR}}-{\rm{B}})\times \frac{{\lambda }_{{\rm{G}}}-{\lambda }_{{\rm{B}}}}{{\lambda }_{{\rm{NIR}}}-{\lambda }_{{\rm{B}}}},$$

The relationship between ABI and TSI based on training data was plotted (Fig. 4b). The fitting results under different water pixel types showed significant correlation with 0.66 (*p* < 0.01, N = 310, Type 1), 0.67 (*p* < 0.01, N = 272, Type 2) and 0.66 (*p* < 0.01, N = 71, Type 3), respectively. The TSI inversion models for different water body types are calculated as slope × ABI + intercept. The values of each coefficient are listed in Table 1.

**Table 1:** Linear fitting coefficients of ABI and TSI based on training dataset.

Lake Type	R^2^	*p*-value	number	slope	intercept
1 (algal-dominated)	0.66	<0.01	310	−601.94	72.47
2 (turbid)	0.67	<0.01	272	−325.78	55.39
3 (clear)	0.66	<0.01	71	−118.28	16.04

## Data Records

The CNLTSI dataset, containing annual average TSI records and intra-lake spatial distribution for 2693 lakes in China, is available at the Zenodo repository (https://zenodo.org/records/11209734)^63^. Lake attributes, including name, location and elevation of lakes, average surface area, corresponding lake zone, row and path where the lake is located, are compiled in ‘*lake_info.csv*’. The long-term yearly TSI records, the 40-year average TSI with trophic levels and trends of TSI variations from 1984–2023 are compiled in ‘*annual_TSI.csv*’. The total of pixel coverage for lakes and annual pixel coverage from 1984–2023 are compiled in ‘*annual_pixel.csv*’. Table 2 displays the field names and corresponding descriptions of the tabular data. Furthermore, the trophic level of 40-year average TSI for each lake were classified and integrated into the table.

**Table 2:** Attribute names of the CNLTSI dataset and the description.

Attribute	Description
Lake ID	Identifies each lake with lake outline dataset.
Lake Name	Lake name acquired from lake outline database. Some lakes have blank names since we cannot define their names.
Lon (DD)	Longitudinal coordinate of the lake’s centroid point in decimal degrees.
Lat (DD)	Latitudinal coordinate of the lake’s centroid point in decimal degrees.
Lake Area	Surface area (km^2^) of the lake derived from lake outline dataset.
Lake Zone	Lake zone in which the lake is located; international lakes are assigned to the lake zone containing the centroid point and may be arbitrary for centroid points falling on the boundaries
Elevation (m)	The average elevation of the lake surface derived from SRTM dataset above sea level.
Row	The row of the Landsat from which TSI was retrieved.
Path	The path of the Landsat from which TSI was retrieved.
TSI_Year	The average TSI on the lake for a given year in the format YYYY. A total of 40-year results were recorded in the column.
Average TSI	The average TSI on the lake from 1984 to 2023.
Trophic Level	The trophic level divided by average TSI on the lake.
Trend_40y	The trend of 40-year TSI for the lake based on Mann-Kendall test.
Slope_40y	The slope of 40-year TSI for the lake based on Mann-Kendall test.
Total Pixels	The total number of pixels representing the lake.
Pixel_Year	The number of TSI pixels for a given year in the format YYYY. A total of 40-year results were recorded in the column.

**Fig. 5:**
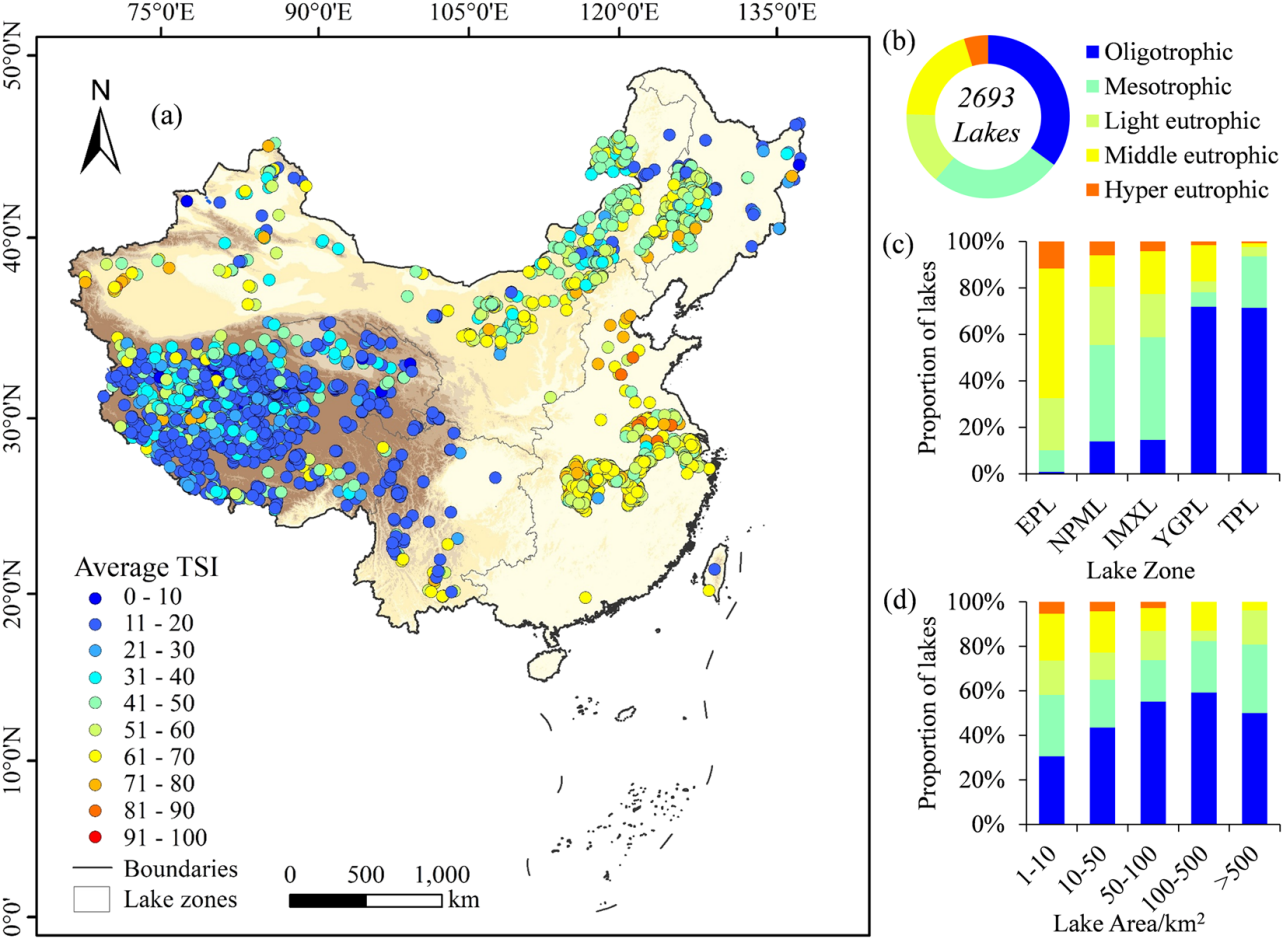
(**a**) Average TSI of lakes in the past 40 years, (**b**) proportions of studied lakes with different trophic levels, and proportions of trophic levels under different (**c**) lake zone and (**d**) lake area.

**Fig. 6:**
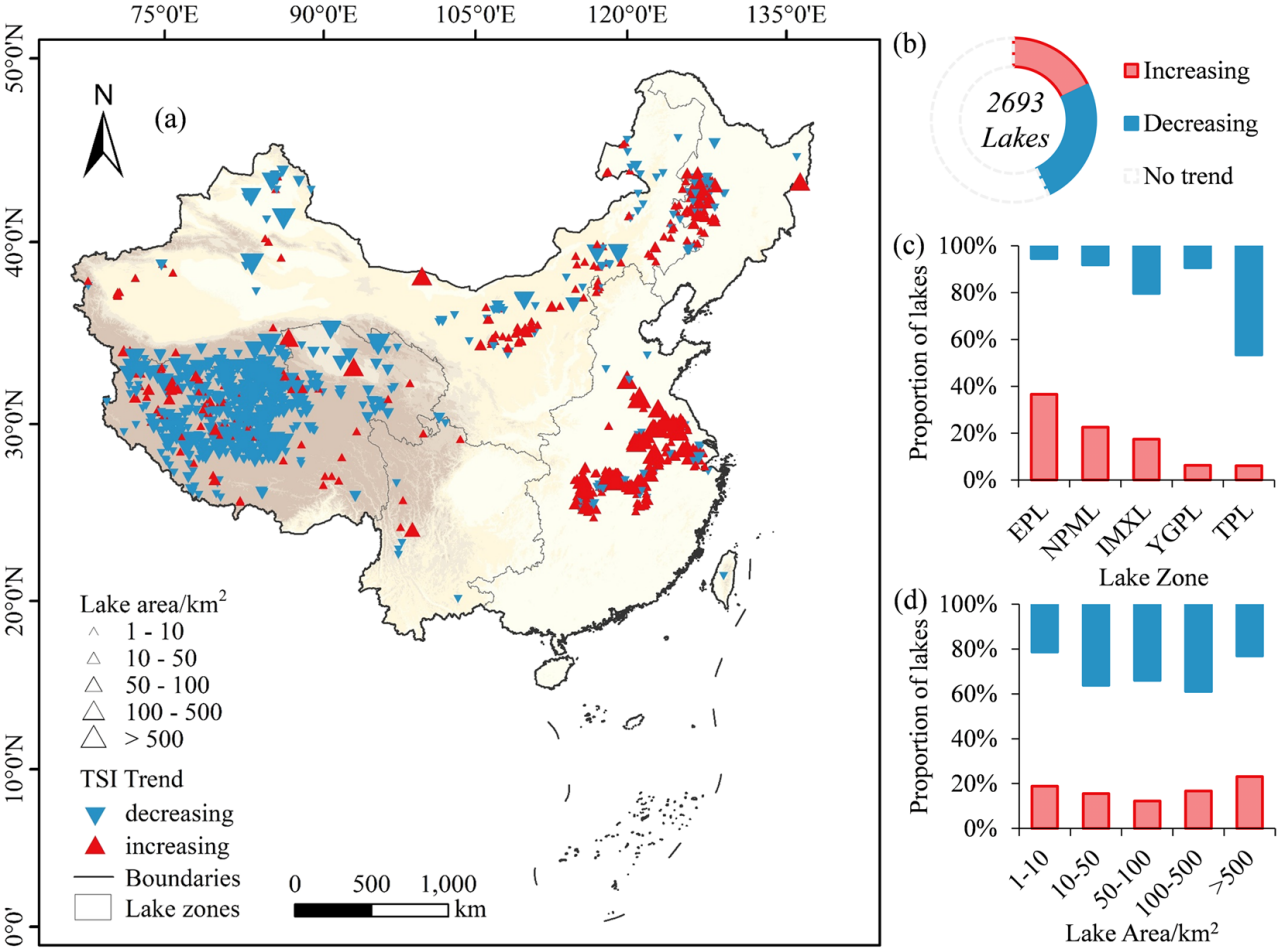
(**a**) Distribution of lakes with significant positive or negative change trend (*p* < 0.01) in the 40 years, (**b**) proportions of studied lakes with different trends, and proportions of lakes with different trends under each (**c**) lake zone and (**d**) lake area.

The raster data of 40-year average TSI for lakes are provided in the ‘*average_TSI*’ folder, with each file named ‘Lake ID_40avg’ and stored in TIFF format. The lake corresponding to each ID can be obtained in ‘*lake_info.csv*’. The average TSI of 2693 lakes in the past four decades is mapped in Fig. 5. Lakes with significant positive or negative change trend (*p < *0.05) in the 40 years are marked in Fig. 6.

## Technical Validation

### Quality of TSI recordings

**Fig. 7:**
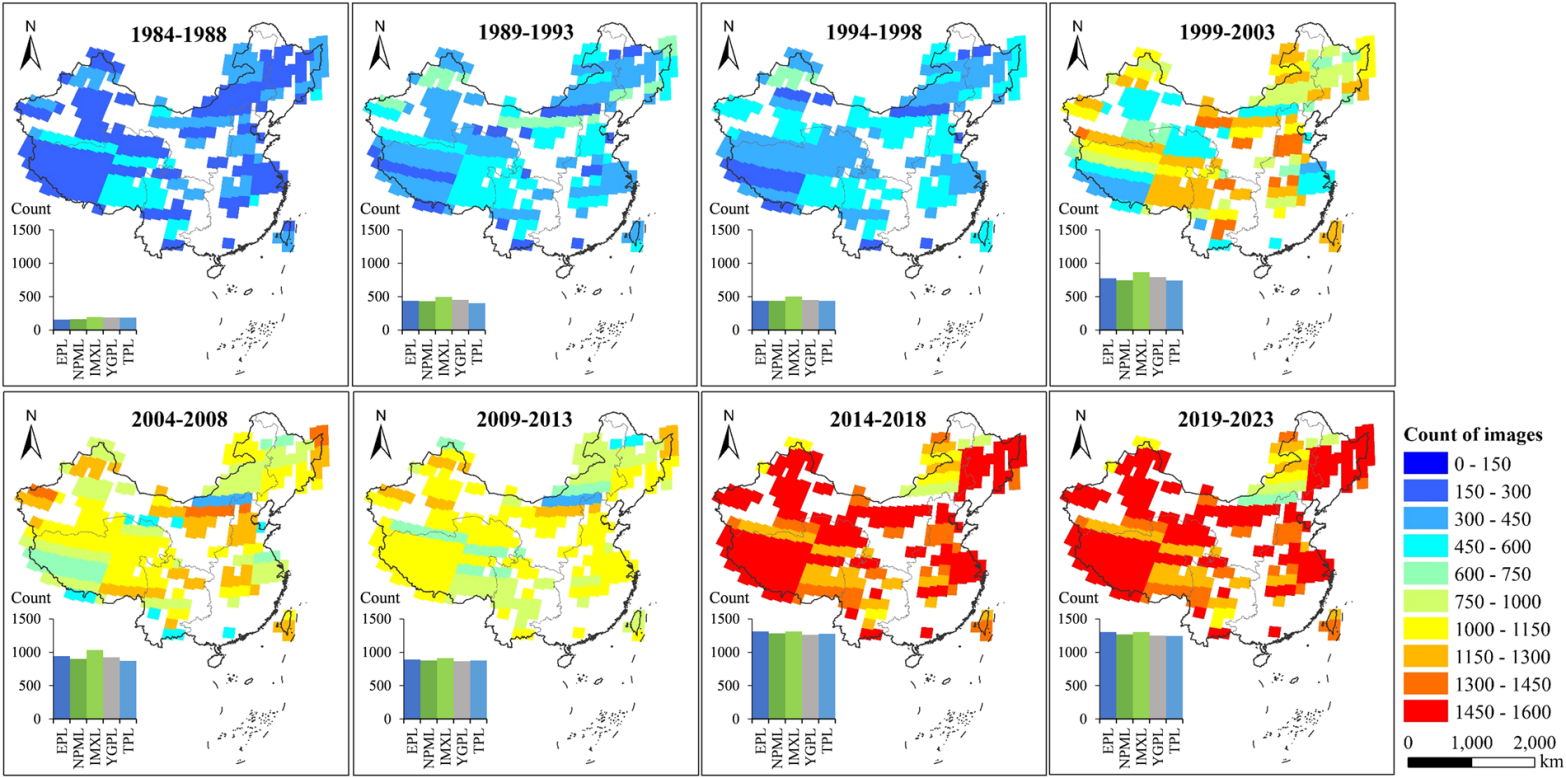
Spatial distributions of the available Landsat observations and numbers of available images in each lake zone from 1984 to 2023 with 5-year intervals.

Figure 7 shows the spatial pattern of all Landsat observations available from 1984 to 2023, with 5-year intervals. The availability of Landsat imagery increased significantly as the monitoring period progressed, owing to the fact that Landsat-8 had greater capacities for on-board recording and satellite-to-ground transmission than previous Landsat systems^64^. In addition, the available Landsat archives before 2000 could not cover the nationwide lakes of China due to the relatively low transmission capabilities, and those for the period before 1988 are particularly limited. The total number of images per map in each lake zone over each 5-year period were also compared (Fig. 7). Lakes in the IMXL and YGPL have consistently higher ranked in terms of observation quantity. The number of available images had evident increasing since 2000 benefited from the joint observation of ETM+ and OLI.

**Fig. 8:**
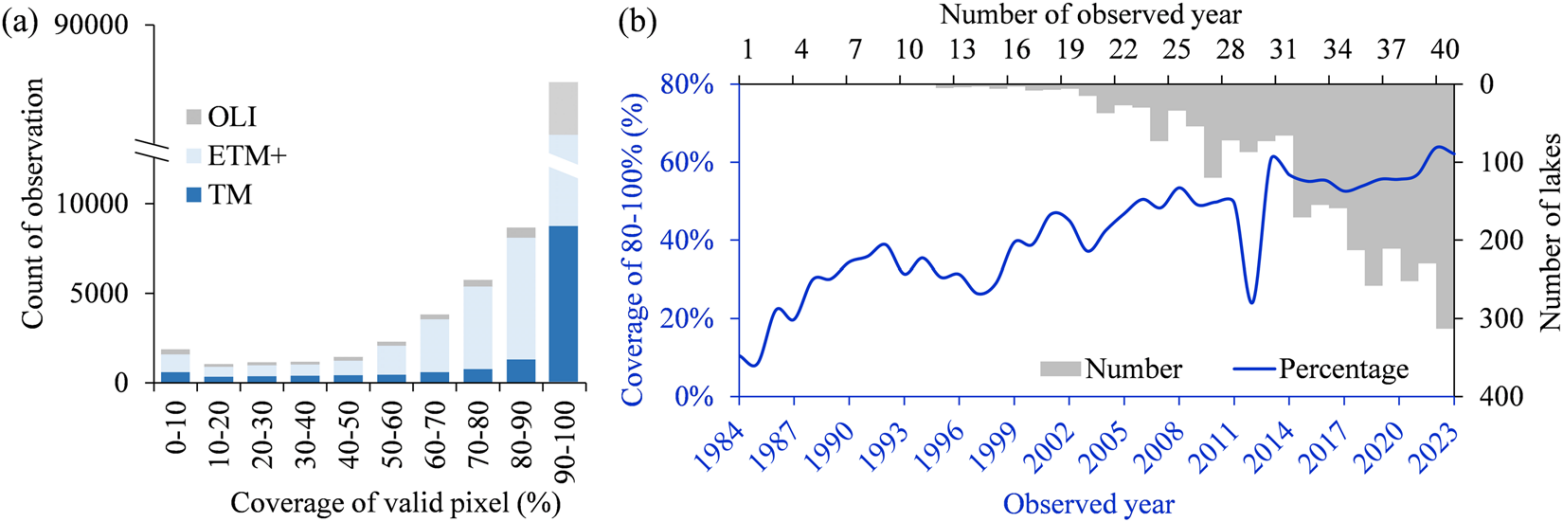
(**a**) Distribution of TSI pixel coverage (%) generated from Landsat imagery, (**b**) yearly percentage of the dataset with TSI coverage ranging from 80%–100% and the average yearly number of available images for lakes in the China.

The coverage of TSI pixel for each image is represented by percentage and counted by the retrieved pixels divided by the total number of pixels for a given lake. Figure 8a shows the distribution of TSI valid pixel coverage based on TM, ETM+ and OLI observations. The proportion with TSI pixel coverage exceeding 50% in the dataset is higher than 92%. The proportion of valid observation with TSI pixel coverage greater than 80% is 83.58%, which was plotted annually in Fig. 8b. Results demonstrated a general increasing in percentage over time, where earlier years (1984–1988) had lower percentages of TSI pixel coverage (<20%). This can be attributed to the incomplete overpass of Landsat-5 before 1988 (Fig. 7). The pixel coverage in 2012 experienced a trough, as only one sensor, Landsat-7 ETM+, was operating under SLC-off issues, resulting in a sharp decline in available pixels. 75.25% lakes had a time series of annual TSI for at least 30 years, and 11.61% of the total lakes in the collection had complete four decades (Fig. 8b). The temporal resolution of our datasets is 16 days from 1984 to 1998 and the year 2012, but shorten to 8 days as two sets of satellites (Landsat-5 and Landsat-7 from 1999 to 2011; Landsat-7 and Landsat-8 from 2013 to 2023) supplied for the generation of the CNLTSI dataset after 1999. Therefore, there was a shorter temporal resolution due to the overlapping transit, leading to inconsistent temporal resolution for the CNLTSI dataset over the past 40 years.

### Validation of lake trophic state

**Fig. 9:**
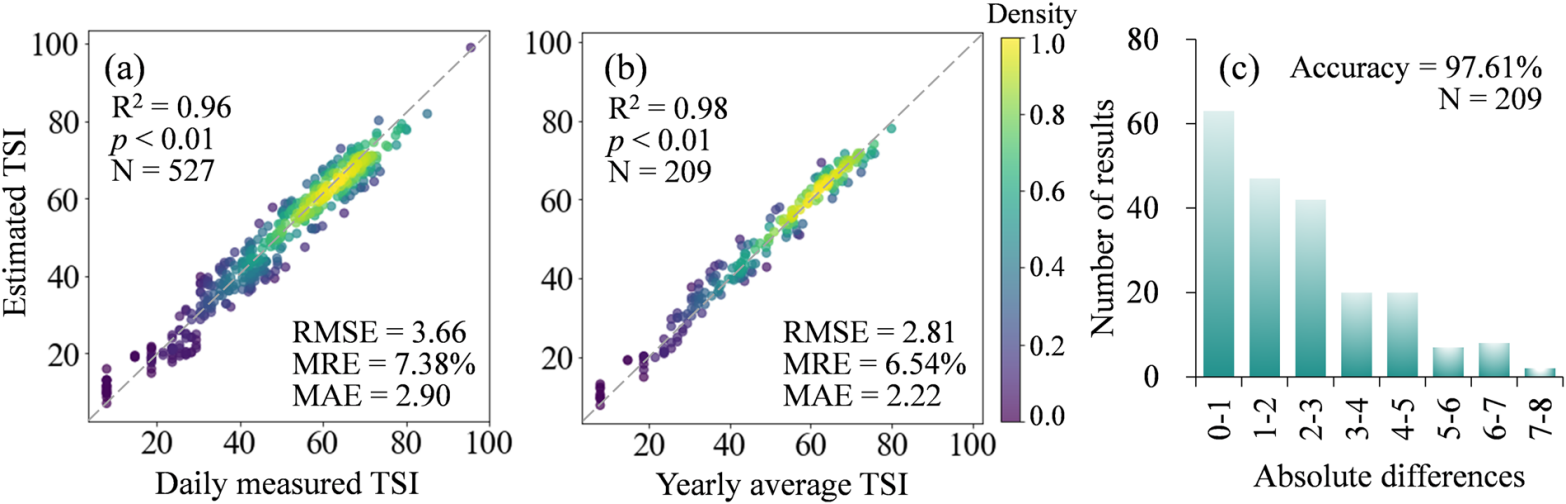
Validation of estimated TSI under (**a**) daily and (**b**) yearly frequency. (**c**) The distribution of absolute differences of yearly estimated TSI were counted.

**Fig. 10:**
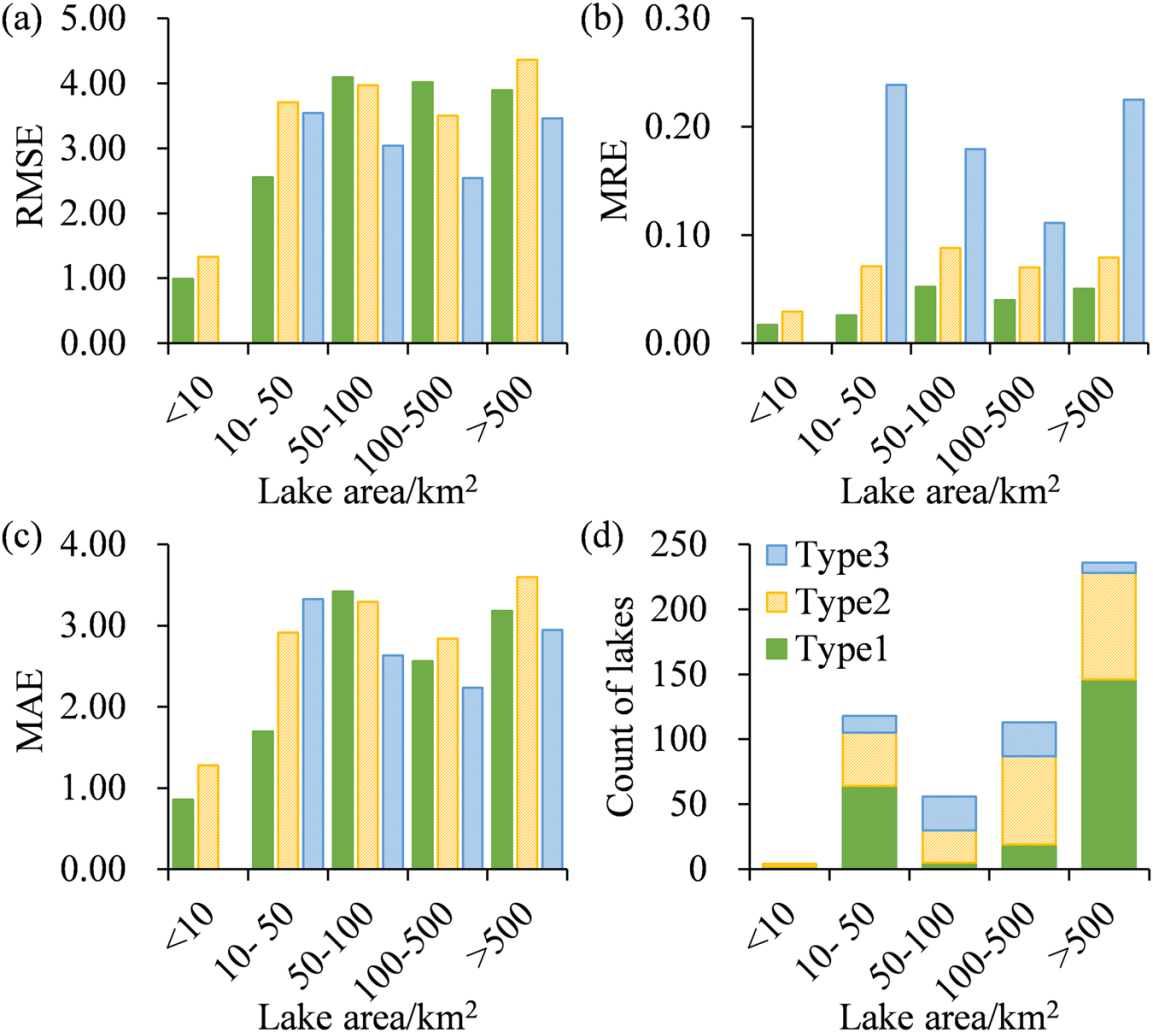
(**a**) RMSE, (**b**) MRE and (**c**) MAE of estimated TSI under different lake area in each water type. (**d**) The count of lakes with different lake sizes in each water type.

The coefficient of determination (R^2^), root mean square error (RMSE), mean relative error (MRE), and mean absolute error (MAE) were used to evaluate the precision of TSI models and CNLTSI results. Statistical parameters, including daily measured TSI calculated from measured Chla and average yearly TSI values from validation dataset, were used to compare against estimated TSI. A good correlation was found between estimated TSI and daily measured TSI (Fig. 9a, R^2^ = 0.96, *p* < 0.01, N = 527). The consistence of estimated TSI under different water types were listed in Table 3, with an overall RMSE of 3.66 and MAE of 2.90. Average yearly TSI was further calculated from these available lakes and showed higher R^2^ of 0.98 for the regression line (*p* < 0.01, N = 209, Fig. 9b). The precision of inversion under different lake area showed that the overall RMSE increased from 1.17 to 4.05 with the increasing of lake sizes (Fig. 10a), which contributed by the algae-dominated water (Type 1) and turbid water pixels (Type 2). The MAE showed the same trend with RMSE (Fig. 10c). In clear water pixels (Type 3), the RMSE of TSI decreasing and 25% lakes (N = 8) were overestimated. The MRE in clear water pixels were higher than that in Type 1 and Type 2 lakes (Fig. 10b). The accuracy of Type 3 water bodies may be limited by the validation samples, resulting in lower performance compared to other water body types (Fig. 10d). At the same time, the construction of the model for clear water pixels relied on the water quality investigation of TPL lakes on the Tibetan Plateau^47^, we matched the images within 3 days to increase the sample size as few TPL lakes concurrent with Landsat overpass. Although the validation of trophic levels showed satisfactory accuracy (>90%), overestimation of TSI resulting from spatiotemporal heterogeneity remain a limitation of this dataset (Fig. 9a).

**Table 3:** Accuracy of daily TSI validation under different water types.

Lake Type	R^2^	*p*-value	RMSE	MRE	MAE	Lake Number
1 (algal-dominated)	0.73	*p* < 0.01	3.58	4.26%	2.71	236
2 (turbid)	0.78	*p* < 0.01	3.92	7.53%	3.18	218
3 (clear)	0.85	*p* < 0.01	3.02	17.05%	2.62	73
All	0.96	*p* < 0.01	3.66	7.38%	2.90	527

These parameters were divided into trophic levels, consistent levels presented a good accuracy of 95.5% (N = 527) in daily validation and 97.6% (N = 209) in yearly validation, respectively (Table 4). Absolute differences between measured TSI and retrieved TSI ranged from 0.01 to 7.37 (Fig. 9c). The number of inversion results with an absolute difference less than 1.0 is the highest, accounting for 30.1% (N = 209, Figs. 9c), and 91.9% of inversion results have an absolute difference less than 5.0.

**Table 4:** Accuracy from different validation datasets.

Dataset	Lake number	Records	Accuracy	Frequency
Field samples	184	527 209	95.5% 97.6%	daily yearly
FUI-derived TSI	125	2112	88.5%	yearly
Environmental Bulletin	29	265	84.2%	yearly
Published papers	52	297	86.5%	yearly

In addition, we verified whether the level calculated from Landsat derived-TSI and validation datasets are consistent within the same time period for validation datasets with only trophic level records, which used as a part of accuracy evaluation. More evaluations were conducted by comparing trophic levels of lakes from FUI-derived TSI dataset, the Chinese Environmental Bulletin and published papers (Table 4). 79.8% of FUI-derived TSI showed consistent trophic level with inversion results (N = 2112). A total of 265 annual records for 29 lakes from the environmental bulletin were further evaluated with an accuracy of 69.1%, and compared 52 more lakes in published papers with an accuracy of 79.4% (N = 297). The overall accuracy of these datasets is more than 80%, illustrating the reliability of the CNLTSI.

### The reliability of the TSI inversions

**Fig. 11:**
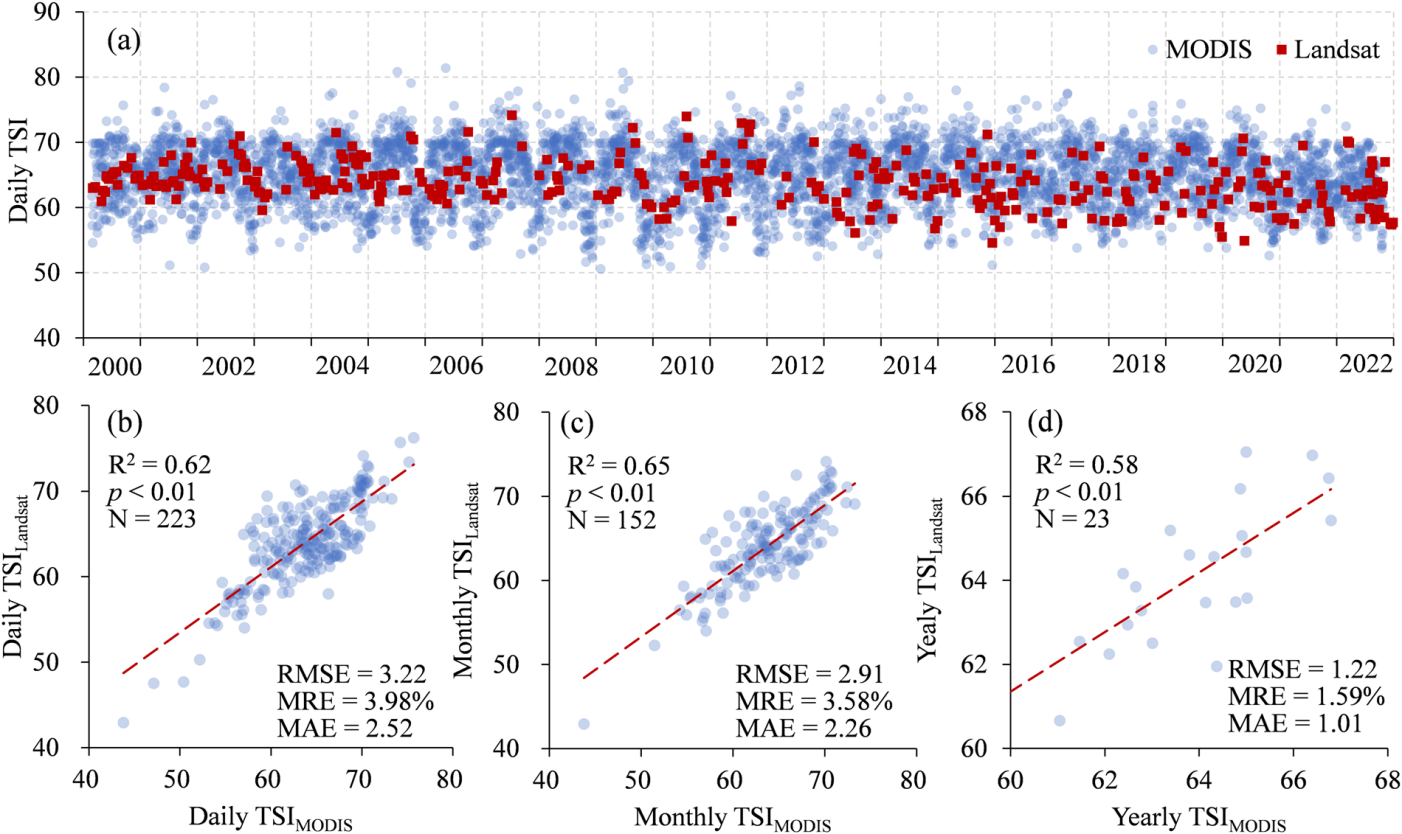
Temporal variation of daily TSI calculated from MODIS and Landsat surface reflectance between 2000 and 2022 (**a**). MODIS-based TSI (TSI_MODIS_) and Landsat-based TSI (TSI_Landsat_) were compared under (**b**) daily, (**c**) monthly and (**d**) yearly frequency.

Despite the 30 m spatial resolution of the Landsat satellites provides the possibility of monitoring small lakes, the 16-day revisit period limits the high-frequency observation for inland waters with the internal mobility the rapid changes^30,65^. Therefore, we evaluated the impact of temporal resolution on the reliability of TSI results. In the evaluation, we included TSI derived from MODIS (1-day revisit, TSI_MODIS_, N = 5079) and compared with Landsat inversions (TSI_Landsat_, N = 420) in the period of 2000 to 2022. Daily MODIS observations can better capture the seasonal changes of trophic state with the mutation of TSI (Fig. 11a), as the standard deviation of TSI_MODIS_ and TSI_Landsat_ is 4.68 and 3.52, respectively.

The daily, monthly and yearly average TSI_Landsat_ and TSI_MODIS_ were further calculated. Daily TSI_Landsat_ records showed high correlation with the concurrent daily TSI_MODIS_ (R^2^ = 0.62, *p* < 0.01, N = 223, Fig. 11b). The correlation increases as the time window expands to inter month (R^2^ = 0.65, *p* < 0.01, N = 72, Fig. 11c), due to the fact that average TSI further weakens the impact on extreme values or mutated values. The consistence of yearly TSI_Landsat_ and TSI_MODIS_ improved, as RMSE and MAE decreases to 1.22 and 1.01, respectively (Fig. 11d). The accuracy of trophic level based on daily, monthly and annual results were as high as 85.2% (N = 223), 89.5% (N = 152) and 100% (N = 23), respectively, indicating the good reliability for the classification of lake trophic level. It should be noted that this study assumes that the TSI results obtained during the 1-day revisit period of MODIS are reliable and ignore the impact of spatial resolution, which can reflect the true situation of water bodies.

**Fig. 12:**
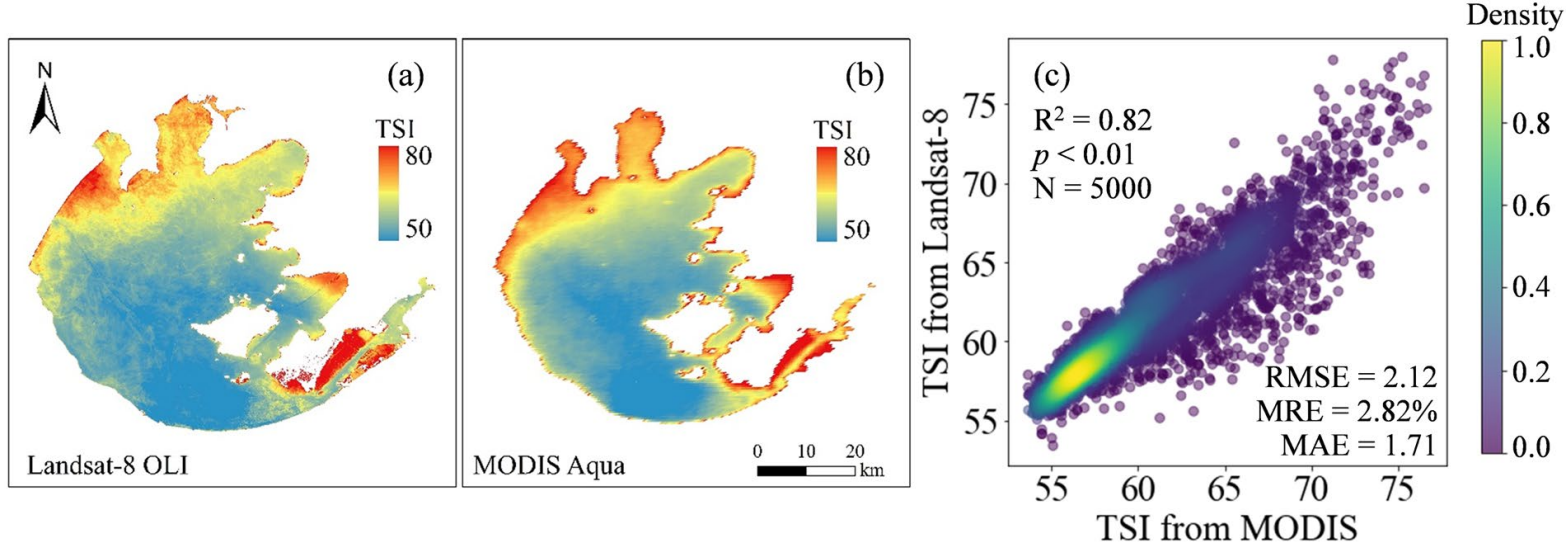
Spatial distribution of average TSI from 2013 to 2020 based on (**a**) Landsat-8 OLI and (**b**) MODIS Aqua images. (**c**) Consistency of TSI derived from MODIS and Landsat-8 were further compared.

The spatial distribution of the average TSI_Landsat_ in Lake Taihu from 2013 to 2020 was further compared with the average TSI_MODIS_ in the same period. TSI pixels were resampled to the same spatial resolution. The spatial distribution of TSI based on Landsat (Fig. 12a) is overall consistent with that of MODIS (Fig. 12b), as the trophic state in the northwest of the Lake Taihu is relatively high, while that of the central and southern region is relatively low. A total of 5000 random points were further generated to extract TSI values corresponding to the position (Fig. 12c). High correlation of 0.82 (*p* < 0.01, N = 5000) was found between TSI_Landsat_ and TSI_MODIS_, with the RMSE and MRE of 2.12 and 2.82%, respectively (Fig. 12c), indicating the satisfactory spatial consistency of TSI results.

## Usage Notes

Lake eutrophication, as one of the major ecological problems, seriously threatens the safety of regional drinking water and harms the ecological environment of lakes. The CNLTSI dataset was released to fill the gap of early monitoring data for small lakes and help to comprehensively analyse the eutrophication process of lakes in China, which provides important reference value for the sustainable development of lakes.

Secondly, the CNLTSI dataset provides a tool for understanding lake quality at the macro-scale. The long time series TSI can be linked to climate factors and anthropogenic activities to explore the driving mechanisms of eutrophication. At the regional scale, the comparison of lakes within the same watershed will promote the understanding of differences between lake scales and eutrophication variations. The intrinsic associations of different lakes within the watershed can be assessed to build systematic perspectives for the watershed eutrophication mechanism. In addition, CNLTSI can be merged with field sampling data to figure out how hydrodynamic, climatic, physicochemical, and biological processes relate to inter-annual variability in trophic state.

Based on the CNLTSI dataset, regional differences in eutrophication rates can provide a basis for water management policies in individual cities, and trends in TSI can verify the deterioration or recovery of lakes under the intervention of human activities. The dataset provides a valuable resource for addressing a variety of basic and applied research questions from local, regional to continental scales.

Considering that users may be interested in understanding the heterogeneity within lakes, i.e., the trophic state of bays or near-shore areas may be different from other areas, the internal spatial of TSI helps to address the above issues. Furthermore, users can extract algal bloom areas and compare the spatial consistency of eutrophic water body pixels with bloom pixels, which helps to assess how seasonal variations in algal growth and the spatial distribution of algae. Our data pipeline allows for automatic re-running of the program, users are able to customise the CNTSI dataset to meet the needs of their specific research questions based on existing infrastructure.

With the launch of Landsat 9, the assessment of trophic state can continue into the future. Our inversion algorithm uses only the visible and near-infrared bands and can therefore be adapted to more satellite sensors. It should be noted that the coefficients of the inversion model need to be adjusted. The construction of virtual constellations in combination with multi satellites is expected to achieve high temporal and spatial resolution observations of the lake eutrophication.

### Supplementary information

SUPPLEMENTARY INFORMATION

## Data Availability

Acquisition and processing of remote sensing images, TSI model construction, and generation of spatial TSI data were done in JavaScript on Google Earth Engine Code Editor and is available at https://github.com/MickyHuu/TSI_inversion.git. The Mann-Kendall-trend was supported by the Python package “pymannkendall” (https://pypi.org/project/pymannkendall), and the significance level (p) was set as 0.05. The visualization of TSI mapping was realized by ArcGIS 10.8.
