# Supplementary material for: A dataset of trophic state index for nation-scale lakes in China from 40-year Landsat observations
Source: Sci Data. 2024 Jun 21;11:659. doi: 10.1038/s41597-024-03506-7 (PMC11192883; doi:10.1038/s41597-024-03506-7)
Supplement: Supplementary file 1 — SUPPLEMENTARY INFORMATION [file 41597_2024_3506_MOESM1_ESM.docx]

**Supplementary Information**

Supplementary Tables S1 – S6

Supplementary Texts 1 – 3

# Table S1 Statistics of water quality parameters of sampled lakes. Oligotrophic, mesotrophic, light eutrophic, moderate eutrophic and hyper eutrophic lakes were abbreviated as OT, MT, LE, ME and HE, respectively. “—” represents no data.

| ID | Lake | Samples | SDD (m) | Chl*a* (µg L^-1^) | SPIM (mg L^-1^) | Level | Zone |
| --- | --- | --- | --- | --- | --- | --- | --- |
| 1 | Weishan | 15 | 0.31±0.15 | 65.25±24.96 | 19.13±18.40 | HE | EPL |
| 2 | Luoma | 9 | 0.59±0.23 | 16.65±7.45 | 12.18±3.24 | LE |  |
| 3 | Taihu | 156 | 0.20±0.14 | 9.12±7.31 | 39.90±20.65 | LE |  |
| 4 | Gehu | 8 | 0.31±0.36 | 28.29±28.04 | 62.10±18.98 | ME |  |
| 5 | Nanyi | 26 | 0.41±0.26 | 56.08±74.67 | 38.51±31.31 | HE |  |
| 6 | Nvshan | 5 | 0.13±0.03 | 93.83±39.29 | 123.75±60.75 | HE |  |
| 7 | Chaohu | 126 | 0.64±0.08 | 40.62±31.42 | 6.73±1.07 | ME |  |
| 8 | Pohu | 12 | 0.29±0.15 | 28.54±27.38 | 33.99±7.53 | ME |  |
| 9 | Huangda | 12 | 0.34±0.04 | 6.75±0.29 | 22.40±1.55 | MT |  |
| 10 | Longgan | 24 | 0.31±0.05 | 64.30±14.73 | 20.00±7.21 | HE |  |
| 11 | Liangzi | 21 | 0.16±.0.09 | 65.55±84.56 | 37.10±26.52 | HE |  |
| 12 | Honghu | 20 | 0.33±0.10 | 44.86±10.13 | 17.39±8.84 | ME |  |
| 13 | Hongze | 65 | 0.62±0.19 | 12.95±5.92 | 12.33±13.08 | LE |  |
| 14 | Gaoyou | 13 | 0.64±0.45 | 21.73±8.20 | 7.67±6.13 | ME |  |
| 15 | Shijiu | 14 | 0.33±0.14 | 64.58±36.99 | 18.00±7.39 | HE |  |
| 16 | Wabu | 5 | 0.51±0.27 | 7.75±4.05 | 28.50±45.64 | LE |  |
| 17 | Caizi | 12 | 0.49±0.10 | 35.72±18.12 | 10.14±6.86 | ME |  |
| 18 | Poyang | 26 | 0.34±0.12 | 74.32±38.48 | 20.32±17.85 | HE |  |
| 19 | Dongting | 26 | 0.74±0.33 | 8.34±7.65 | 11.85±7.12 | LE |  |
| 20 | Erhai | 75 | 1.92±0.48 | 9.76±5.33 | 2.22±2.46 | MT | YGPL |
| 21 | Qilu | 12 | 0.39±0.04 | 125.06±15.72 | 2.60±2.62 | HE |  |
| 22 | Xingyun | 88 | 0.58±0.09 | 68.99±64.46 | 2.12±1.75 | HE |  |
| 23 | Dianchi | 12 | 0.27±0.05 | 71.50±7.96 | — | HE |  |
| 24 | Fuxian | 12 | 6.23±0.76 | 0.61±0.33 | — | OT |  |
| 25 | Wuliangsu | 90 | 1.38±0.42 | 6.16±4.31 | 8.30±3.92 | MT | IMXL |
| 26 | Daihai | 18 | 2.22±0.52 | 1.53±0.67 | 64.62±15.08 | MT |  |
| 27 | Hulun | 15 | 0.29±0.03 | 21.93±10.77 | 23.11±9.66 | ME |  |
| 28 | Chagan | 21 | 3.69±0.48 | 21.97±7.16 | 15.49±7.56 | ME | NPML |
| 29 | Urru Co | 1 | — | 0.10 | — | OT | TPL |
| 30 | Yamdrok | 1 | — | 0.10 | — | OT |  |
| 31 | Cam Co | 1 | — | 0.30 | — | OT |  |
| 32 | Gomang Co | 1 | — | 0.30 | — | OT |  |
| 33 | Dagze | 1 | — | 0.50 | — | OT |  |
| 34 | Bangong | 1 | — | 0.50 | — | OT |  |
| 35 | Cona | 1 | — | 0.60 | — | OT |  |
| 36 | Hoh Xil | 1 | — | 0.70 | — | OT |  |
| 37 | Salt Lake | 1 | — | 0.90 | — | OT |  |

# Table S2 Field sampled lakes for validation of TSI

| ID | Lake name | LON. | LAT. | Observe year |
| --- | --- | --- | --- | --- |
| 1 | Aksai Chin Lake | 79.79 | 35.25 | 2015 |
| 2 | Ang Laren Lake | 79.79 | 35.25 | 2015 |
| 3 | Angda'er Co | 83.04 | 31.57 | 2017 |
| 4 | Angrenjin Co | 89.57 | 32.70 | 2020 |
| 5 | A'ong Co | 87.19 | 29.30 | 2018 |
| 6 | Baidoi Co | 81.76 | 32.75 | 2012 |
| 7 | Bam Co | 87.82 | 32.79 | 2020 |
| 8 | Bangda Lake | 90.62 | 31.26 | 2018 |
| 9 | Bangong Lake | 81.53 | 34.96 | 2015 |
| 10 | Baosai'er Co | 79.81 | 33.55 | 2017 |
| 11 | Beileicuo | 89.43 | 32.34 | 2017 |
| 12 | Bero Zeco | 88.41 | 32.93 | 2020 |
| 13 | Bingni | 82.96 | 32.44 | 2018 |
| 14 | Burog Co | 86.31 | 30.61 | 2018 |
| 15 | Caiji Co | 85.44 | 31.21 | 2012 |
| 16 | Cam Co | 83.55 | 32.11 | 2018 |
| 17 | Chaohu | 117.51 | 31.52 | 2013-2016 |
| 18 | Chibzhang Co | 90.23 | 33.38 | 2016 |
| 19 | Co Ngoin | 91.51 | 31.49 | 2018 |
| 20 | Cona | 91.51 | 32.05 | 2018 |
| 21 | Caizi | 117.10 | 30.79 | 2018 |
| 22 | Dagze Lake | 87.54 | 31.86 | 2017 |
| 23 | Dajia Lake | 85.73 | 29.81 | 2018 |
| 24 | Dangqiong Co | 86.80 | 31.56 | 2018 |
| 25 | Darao Co | 83.21 | 32.48 | 2018 |
| 26 | Daru Co | 90.74 | 31.67 | 2017 |
| 27 | Dawa Lake | 84.96 | 31.27 | 2018 |
| 28 | Daxiong Lake | 85.60 | 34.06 | 2013 |
| 29 | Dianchi | 102.70 | 24.84 | 2019 |
| 30 | Deru Co | 88.91 | 32.67 | 2016 |
| 31 | Dibu Co | 84.82 | 30.79 | 2012 |
| 32 | Dogai Coring | 88.98 | 34.55 | 2016 |
| 33 | Dogaicorng Qangco | 35.31 | 89.29 | 2012 |
| 34 | Dongbu Co | 87.21 | 31.28 | 2018 |
| 35 | Dorsoidong Co | 89.83 | 33.35 | 2016 |
| 36 | Dongting | 113.15 | 29.43 | 2018 |
| 37 | Dung Co | 91.17 | 31.67 | 2018 |
| 38 | Erhai | 100.18 | 25.87 | 2019 |
| 39 | Ga'a Co | 88.96 | 32.20 | 2017 |
| 40 | Garen Co | 84.98 | 30.79 | 2018 |
| 41 | Gemang Co | 87.28 | 31.59 | 2018 |
| 42 | Gomang Co | 89.20 | 31.21 | 2019 |
| 43 | Gozha Co | 81.06 | 35.02 | 2015 |
| 44 | Guogen Co | 89.19 | 32.40 | 2017 |
| 45 | Gaoyou | 119.19 | 32.80 | 2017 |
| 46 | Gyaring Co | 88.49 | 31.02 | 2017 |
| 47 | Huangda | 116.31 | 30.03 | 2018 |
| 48 | Hoh Xil Lake | 91.24 | 35.58 | 2019 |
| 49 | Hongze | 118.83 | 33.34 | 2016- 2018 |
| 50 | Jiang Co | 90.84 | 31.53 | 2017 |
| 51 | Jieze Caka | 80.88 | 33.94 | 2018 |
| 52 | Jingyu Lake | 89.43 | 36.33 | 2020 |
| 53 | Kunggyu Co | 82.12 | 30.63 | 2017 |
| 54 | Kyebxang Co | 89.95 | 32.45 | 2019 |
| 55 | Lagkor Co | 84.14 | 32.04 | 2018 |
| 56 | Lake Aqqikkol | 88.41 | 37.09 | 2020 |
| 57 | Lake Ayakum | 89.48 | 37.55 | 2020 |
| 58 | Lake Jargö | 88.22 | 31.82 | 2019 |
| 59 | Lake Manasarovar | 81.44 | 30.64 | 2017 |
| 60 | Lake Rakshastal | 81.25 | 30.67 | 2017 |
| 61 | Laxiong Co | 85.21 | 34.35 | 2013 |
| 62 | Lexie Wudan Lake | 90.20 | 35.74 | 2019 |
| 63 | Luoma | 118.23 | 34.06 | 2017 |
| 64 | Longmu Lake | 80.47 | 34.62 | 2015 |
| 65 | Longwe Lake | 88.31 | 33.86 | 2012 |
| 66 | Lumajangdong Co | 81.68 | 34.01 | 2018 |
| 67 | Ma'erxia Co | 87.55 | 30.95 | 2018 |
| 68 | Mang Co | 80.45 | 34.51 | 2015 |
| 69 | Mucuo | 86.23 | 30.66 | 2018 |
| 70 | Mudidalayu Co | 88.61 | 30.58 | 2019 |
| 71 | Mugqu Co | 89.01 | 31.04 | 2019 |
| 72 | Nam Co | 90.78 | 30.77 | 2016 |
| 73 | Neri Pumco | 91.48 | 31.30 | 2018 |
| 74 | Ngangze Co | 87.13 | 31.03 | 2018 |
| 75 | Norma Co | 88.06 | 32.40 | 2019 |
| 76 | Nansi | 118.10 | 32.99 | 2017 |
| 77 | Pohu | 116.43 | 30.15 | 2017 |
| 78 | Pongyin Co | 88.21 | 32.90 | 2020 |
| 79 | Pung Co | 90.93 | 31.50 | 2018 |
| 80 | Poyang | 116.06 | 29.37 | 2010 |
| 81 | Qige Co | 85.52 | 31.20 | 2012 |
| 82 | Quanshui Lake | 80.15 | 34.75 | 2015 |
| 83 | Rebang Co | 80.53 | 33.04 | 2017 |
| 84 | Rengqingxiubu Lake | 83.47 | 31.28 | 2017 |
| 85 | Salt Lake | 93.43 | 35.50 | 2019 |
| 86 | Serbug Co | 88.23 | 32.01 | 2019 |
| 87 | Siling Co | 88.98 | 31.75 | 2014 |
| 88 | Songmuxi Co | 80.23 | 34.59 | 2012 |
| 89 | Taihu | 120.19 | 31.48 | 2004, 2009, 2010, 2013, 2014 |
| 90 | Taiyang Lake | 90.61 | 35.90 | 2019 |
| 91 | Tangra Yumco | 86.64 | 31.26 | 2018 |
| 92 | Taruo Lake | 84.14 | 31.14 | 2014 |
| 93 | Telashi Lake | 92.24 | 34.81 | 2019 |
| 94 | Tong Tso | 84.77 | 32.16 | 2018 |
| 95 | Tso Ngon | 88.76 | 31.62 | 2017 |
| 96 | Urru Co | 87.93 | 31.71 | 2019 |
| 97 | Wulanwula Lake | 90.67 | 34.77 | 2019 |
| 98 | Wuma Co | 83.18 | 32.44 | 2018 |
| 99 | Xijir Ulan Lake | 90.33 | 35.21 | 2019 |
| 100 | Xinxing Lake | 80.75 | 34.79 | 2015 |
| 101 | Xueyuan Lake | 85.71 | 34.29 | 2013 |
| 102 | Xuru Co | 86.42 | 30.31 | 2013 |
| 103 | Xingyun | 102.79 | 24.36 | 2009 |
| 104 | Yagain Co | 89.80 | 33.04 | 2020 |
| 105 | Yahu Lake | 89.07 | 33.18 | 2020 |
| 106 | Yamdrok Lake | 90.87 | 28.95 | 2019 |
| 107 | Yibug Caka | 86.71 | 32.97 | 2020 |
| 108 | Yoqag Co | 88.62 | 30.48 | 2019 |
| 109 | Zhangnai Co | 87.41 | 31.55 | 2012 |
| 110 | Zhen Co | 90.53 | 28.97 | 2019 |
| 111 | Zhuonai Lake | 91.94 | 35.56 | 2020 |
| 112 | Zigetang Co | 90.85 | 32.05 | 2017 |

# Table S3 Information of lakes from the investigation report in China (2019) for validation.

| ID | LON | LAT | Lake name | ID | LON | LAT | Lake name |
| --- | --- | --- | --- | --- | --- | --- | --- |
| 1 | 116.68 | 32.33 | Anfeng | 38 | 118.21 | 34.05 | Luoma |
| 2 | 117.4 | 30.8 | Baidang | 39 | 111.95 | 29.42 | Maoli |
| 3 | 119.12 | 33.29 | Baima | 40 | 116.21 | 29.68 | Nanbei |
| 4 | 115.99 | 38.91 | Baiyangdian | 41 | 117.32 | 34.6 | Nansi |
| 5 | 114.73 | 30.26 | Baoan | 42 | 118.98 | 31.14 | Nanyi |
| 6 | 111.88 | 29.72 | Beimin | 43 | 111.97 | 29.84 | Niulang |
| 7 | 117.12 | 30.79 | Caizi | 44 | 118.11 | 33.01 | Nvshan |
| 8 | 112.44 | 30.45 | Chang | 45 | 117.18 | 30.67 | Pogang |
| 9 | 117.48 | 31.54 | Chaohu | 46 | 116.09 | 29.47 | Poyang |
| 10 | 120.81 | 31.2 | Cheng | 47 | 114.76 | 30.32 | Sanshan |
| 11 | 116.26 | 32.36 | Chengxi | 48 | 112.02 | 29.44 | Shanbo |
| 12 | 116.38 | 28.66 | Chenjia | 49 | 112.44 | 30.45 | Shangjin |
| 13 | 115.39 | 30.14 | Cidong | 50 | 114.21 | 30.13 | Shangshe |
| 14 | 115.06 | 30.22 | Cihu | 51 | 119.48 | 32.54 | Shaobo |
| 15 | 112.52 | 29.24 | Datong | 52 | 117.04 | 30.38 | Shengjin |
| 16 | 115.09 | 30.1 | Daye | 53 | 115.81 | 29.99 | Taibai |
| 17 | 120.92 | 31.09 | Dianshan | 54 | 116.75 | 30.02 | Taibo |
| 18 | 116.2 | 36.05 | Dongping | 55 | 120.19 | 31.2 | Taihu |
| 19 | 121.68 | 29.77 | Dongqian | 56 | 114.38 | 30.4 | Tangxun |
| 20 | 112.48 | 28.86 | Dongting | 57 | 114.17 | 30.81 | Tongjia |
| 21 | 113.66 | 30.81 | Dongxicha | 58 | 116.87 | 32.46 | Wabu |
| 22 | 114.2 | 30.05 | Futou | 59 | 115.34 | 29.87 | Wanghu |
| 23 | 117.14 | 32.71 | Gaotang | 60 | 116.68 | 30.27 | Wuchang |
| 24 | 119.41 | 32.8 | Gaoyou | 61 | 115.58 | 29.92 | Wushan |
| 25 | 119.85 | 31.67 | Gehu | 62 | 117.7 | 33.12 | Xiangjian |
| 26 | 115.24 | 30.06 | Haikou | 63 | 116.15 | 29.37 | Xinmiao |
| 27 | 115.64 | 37.65 | Hengshui | 64 | 120.74 | 31.45 | Yangcheng |
| 28 | 113.43 | 29.92 | Honghu | 65 | 114.47 | 30.57 | Yanxi |
| 29 | 118.8 | 33.29 | Hongze | 66 | 116.07 | 28.71 | Yaohu |
| 30 | 116.34 | 30.05 | Huangda | 67 | 114.08 | 30.88 | Yezhu |
| 31 | 113.56 | 29.74 | Huanggai | 68 | 119.05 | 32.8 | Yihu |
| 32 | 116.31 | 28.6 | Junshan | 69 | 120.9 | 31.07 | Yuandang |
| 33 | 120.75 | 31.6 | Kuncheng | 70 | 112.08 | 30.06 | Yuhu |
| 34 | 114.46 | 30.24 | Liangzi | 71 | 112.1 | 29.8 | Yuni |
| 35 | 111.74 | 29.1 | Liuye | 72 | 114.69 | 30.65 | Zhangdu |
| 36 | 116.11 | 29.92 | Longgan | 73 | 116.67 | 29.15 | Zhuhu |
| 37 | 114.21 | 30.22 | Luhu |  |  |  |  |

# Table S4 Lakes from the FUI-derived TSI dataset in China for validation.

| ID | LON | LAT | Lake_name | Year |
| --- | --- | --- | --- | --- |
| 1 | 82.89 | 44.92 | Ebi | 2000-2018 |
| 2 | 81.16 | 44.60 | Sai li-mu | 2005-2018 |
| 3 | 87.29 | 47.28 | Ulungur | 2000-2018 |
| 4 | 87.37 | 47.00 | Jili | 2000-2018 |
| 5 | 86.75 | 41.83 | Bosten | 2000-2018 |
| 6 | 78.76 | 39.79 | Xiaoxihaiz Shuiku | 2005-2018 |
| 7 | 80.48 | 34.62 | Lung Mu | 2007-2018 |
| 8 | 81.87 | 34.45 |  | 2007-2018 |
| 9 | 82.25 | 34.26 | Memar | 2007-2018 |
| 10 | 79.79 | 34.17 | Chem | 2000, 2001, 2007-2018 |
| 11 | 81.64 | 34.05 | Lumajangdong | 2000, 2007-2018 |
| 12 | 80.90 | 33.95 | Gyeze Caka | 2001, 2007-2018 |
| 13 | 82.31 | 34.11 | Aru | 2000, 2007-2018 |
| 14 | 84.64 | 35.43 |  | 2003, 2007-2018 |
| 15 | 79.03 | 33.75 | Bangong | 2000, 2001, 2007-2018 |
| 16 | 81.74 | 34.52 |  | 2007-2018 |
| 17 | 81.23 | 30.85 | La-ang | 2001, 2007-2018 |
| 18 | 83.13 | 35.29 |  | 2001, 2006-2018 |
| 19 | 79.82 | 35.22 | Aksayquin | 2000, 2001, 2008-2018 |
| 20 | 83.11 | 35.02 | Bairab | 2001, 2006-2018 |
| 21 | 81.08 | 35.02 | Gozha | 2007-2018 |
| 22 | 81.56 | 34.95 | Bangdag | 2007-2018 |
| 23 | 81.89 | 34.74 |  | 2007, 2008, 2010-2018 |
| 24 | 79.69 | 34.68 | Surigh Yilganing Kol | 2008-2018 |
| 25 | 117.71 | 49.37 | Hulun | 2000-2018 |
| 26 | 93.87 | 38.86 | Suhai | 2000-2018 |
| 27 | 88.41 | 37.08 | Aqqikkol | 2000-2018 |
| 28 | 86.41 | 30.31 | Hsu-ju | 2001, 2004-2018 |
| 29 | 84.80 | 30.79 |  | 2001, 2004-2018 |
| 30 | 95.16 | 37.02 | Dabsan | 2001, 2004-2018 |
| 31 | 89.55 | 36.27 | Arkatag | 2002, 2003, 2005, 2010-2018 |
| 32 | 90.72 | 35.93 |  | 2000, 2001, 2003-2018 |
| 33 | 92.91 | 35.70 | Hoh Sai | 2000-2018 |
| 34 | 90.21 | 35.74 | Lixi'Oidain | 2000-2018 |
| 35 | 97.56 | 38.30 | Hala | 2000-2018 |
| 36 | 92.11 | 35.53 | Zonag | 2000-2018 |
| 37 | 88.38 | 35.41 |  | 2000-2018 |
| 38 | 89.26 | 35.32 | Dogaicoring Quang | 2000-2018 |
| 39 | 90.78 | 35.63 |  | 2000-2018 |
| 40 | 90.33 | 35.21 | Xijir Ulan | 2000-2018 |
| 41 | 92.29 | 35.21 | Dorge | 2000-2018 |
| 42 | 86.76 | 35.12 |  | 2003, 2004, 2006-2018 |
| 43 | 90.52 | 35.06 |  | 2000-2018 |
| 44 | 97.31 | 34.81 | Gyaring | 2000-2018 |
| 45 | 90.50 | 34.71 |  | 2000-2012, 2014-2018 |
| 46 | 89.00 | 34.55 | Dogai Coring | 2000-2018 |
| 47 | 85.22 | 34.35 |  | 2006-2018 |
| 48 | 87.04 | 33.88 |  | 2003, 2004, 2006-2018 |
| 49 | 89.43 | 37.56 | Ayakkum | 2000-2018 |
| 50 | 91.20 | 33.89 |  | 2000-2018 |
| 51 | 88.60 | 33.86 |  | 2000-2018 |
| 52 | 90.36 | 33.48 | Migriggyangzham | 2000-2018 |
| 53 | 89.87 | 33.44 | Dorsoidong | 2000-2018 |
| 54 | 89.81 | 33.00 | Yaggain Canco | 2001, 2004-2018 |
| 55 | 88.70 | 32.98 |  | 2000-2018 |
| 56 | 88.03 | 32.37 |  | 2000-2018 |
| 57 | 84.75 | 32.17 | Dong | 2000-2018 |
| 58 | 83.56 | 32.11 | Cam | 2007-2018 |
| 59 | 90.84 | 32.08 | Namru | 2000-2018 |
| 60 | 89.05 | 31.78 | Siling | 2004-2018 |
| 61 | 91.51 | 31.92 | Na | 2000-2018 |
| 62 | 84.14 | 32.02 | Lagkor | 2001, 2004, 2007-2018 |
| 63 | 87.94 | 31.70 | Dagze | 2004-2018 |
| 64 | 90.40 | 31.78 |  | 2000-2018 |
| 65 | 88.04 | 31.30 | Ko-jen | 2004-2018 |
| 66 | 89.51 | 31.73 | Bangkog | 2004-2018 |
| 67 | 83.05 | 31.57 | Ngangla Ringco | 2008-2018 |
| 68 | 87.23 | 31.60 |  | 2004, 2004-2018 |
| 69 | 90.97 | 31.51 | P'eng-Ts'o | 2000-2018 |
| 70 | 87.96 | 31.39 |  | 2004-2018 |
| 71 | 86.50 | 30.95 | Tangra | 2001, 2004-2018 |
| 72 | 90.59 | 31.27 | Pa-Mu-Ts'o | 2000-2018 |
| 73 | 84.05 | 31.43 | Cha-pu-yeh ch'a-k'a | 2001, 2012-2018 |
| 74 | 83.46 | 31.26 | Jen-ch'ing hsiu-pu-ts'o | 2001, 2004-2018 |
| 75 | 89.20 | 31.21 | Kuo-mang | 2004-2018 |
| 76 | 84.95 | 31.24 |  | 2001, 2004, 2008-2018 |
| 77 | 91.04 | 31.27 | Bong | 2000-2018 |
| 78 | 84.01 | 30.53 | Tajo-Tzo | 2000, 2001, 2005-2018 |
| 79 | 87.15 | 31.04 | Ang-tzu | 2001, 2004-2018 |
| 80 | 85.64 | 30.95 | Zhari Namco | 2001, 2004-2018 |
| 81 | 96.92 | 37.13 | Toson | 2000-2018 |
| 82 | 83.57 | 30.88 | P'a-lung | 2001, 2004-2018 |
| 83 | 89.78 | 30.91 |  | 2007-2018 |
| 84 | 90.66 | 30.71 | Nam | 2000-2018 |
| 85 | 84.80 | 30.21 |  | 2001, 2004-2018 |
| 86 | 81.37 | 30.76 | Ma-p'ang yung-ts'o | 2001, 2008-2018 |
| 87 | 86.29 | 30.64 | Mu-ts'o-ping-ni | 2001, 2004-2018 |
| 88 | 85.73 | 29.84 |  | 2001, 2004-2018 |
| 89 | 90.80 | 38.13 | Gas Hure | 2000-2018 |
| 90 | 85.61 | 28.86 | P'ei-k'u t'so | 2001-2018 |
| 91 | 90.38 | 29.10 | Yamdrok | 2000-2018 |
| 92 | 124.04 | 45.72 | Yueliang Pao | 2000-2018 |
| 93 | 124.41 | 45.29 | Chagan | 2000-2018 |
| 94 | 116.66 | 43.34 | Dalai | 2000-2018 |
| 95 | 112.69 | 40.57 | Daihai | 2000-2018 |
| 96 | 100.20 | 36.92 | Qinghai | 2000-2018 |
| 97 | 98.38 | 35.35 | Donggi Cona | 2001, 2004-2018 |
| 98 | 97.78 | 35.11 | Ngoring | 2000-2018 |
| 99 | 116.24 | 32.38 |  | 2000-2018 |
| 100 | 116.42 | 32.39 | Chengdong | 2000-2018 |
| 101 | 112.82 | 30.40 | Chang | 2000-2018 |
| 102 | 114.60 | 30.26 | Liangzi | 2000-2018 |
| 103 | 114.18 | 30.07 | Futou | 2000-2018 |
| 104 | 116.21 | 36.12 | Dongping | 2000-2018 |
| 105 | 116.98 | 34.87 | Weishan | 2000-2018 |
| 106 | 118.31 | 34.12 | Luoma | 2000-2018 |
| 107 | 118.73 | 33.09 | Hungtze | 2000-2018 |
| 108 | 113.47 | 29.92 | Hong | 2000-2018 |
| 109 | 112.99 | 29.35 | Dongting | 2000-2018 |
| 110 | 100.66 | 26.55 | Ch'eng | 2000-2018 |
| 111 | 100.22 | 25.64 | Erh | 2000-2018 |
| 112 | 102.60 | 24.78 | Dian Chi | 2000-2018 |
| 113 | 102.94 | 24.52 | Fuxian | 2000-2018 |
| 114 | 118.84 | 31.11 | Nanyi | 2000-2018 |
| 115 | 116.50 | 30.14 | Po | 2000-2018 |
| 116 | 116.47 | 30.08 | Daguan | 2000-2018 |
| 117 | 119.46 | 32.56 | Kaoyu | 2000-2018 |
| 118 | 119.78 | 31.49 | Ge | 2000-2018 |
| 119 | 119.51 | 31.55 | Chang Dang | 2000-2018 |
| 120 | 117.85 | 31.59 | Chao | 2000-2018 |
| 121 | 118.77 | 31.49 | Shijiu | 2000-2018 |
| 122 | 120.54 | 31.11 | Tai | 2000-2018 |
| 123 | 120.83 | 31.39 | Yangcheng | 2000-2018 |
| 124 | 116.26 | 30.01 | Longgan | 2000-2018 |
| 125 | 116.22 | 29.75 | Poyang | 2000-2018 |

# Table S5 Lakes reported by the China Environmental Bullet for validation.

| ID | LON. | LAT. | Lake name | Year |
| --- | --- | --- | --- | --- |
| 1 | 119.14 | 33.27 | Baima | 2016-2020 |
| 2 | 115.96 | 38.87 | Baiyangdian | 2005-2019 |
| 3 | 117.07 | 30.8 | Caizi | 2012-2019 |
| 4 | 117.53 | 31.57 | Chaohu | 1995, 1999, 2001-2019 |
| 5 | 113.62 | 30.04 | Datong | 2016-2019 |
| 6 | 120.96 | 31.12 | Dianshan | 2012-2019 |
| 7 | 114.4 | 30.56 | Donghu | 2006-2011 |
| 8 | 116.2 | 35.98 | Dongping | 2012-2019 |
| 9 | 121.66 | 29.77 | Dongqian | 2016-2019 |
| 10 | 112.74 | 29.07 | Dongting | 2003-2019 |
| 11 | 114.23 | 30.02 | Futou | 2012-2019 |
| 12 | 86.67 | 33.2 | Gaotang | 2017-2019 |
| 13 | 119.29 | 32.85 | Gaoyou | 2012-2019 |
| 14 | 115.6 | 37.61 | Hengshui | 2016-2019 |
| 15 | 113.34 | 29.86 | Honghu | 2012-2019 |
| 16 | 118.59 | 33.31 | Hongze | 2009-2019 |
| 17 | 116.38 | 30.02 | Huangda | 2016-2021 |
| 18 | 116.6 | 32.59 | Jiaogang | 2016-2022 |
| 19 | 114.51 | 30.23 | Liangzi | 2012-2019 |
| 20 | 116.15 | 29.95 | Longgan | 2012-2019 |
| 21 | 118.19 | 34.11 | Luoma | 2012-2019 |
| 22 | 116.96 | 34.87 | Nansi | 2003-2019 |
| 23 | 118.96 | 31.11 | Nanyi | 2012-2019 |
| 24 | 116.28 | 29.11 | Poyang | 2003-2019 |
| 25 | 112.5 | 29.64 | Shengjin | 2012-2019 |
| 26 | 120.19 | 31.2 | Taihu | 1995, 1999, 2001-2019 |
| 27 | 116.89 | 32.4 | Wabu | 2012-2019 |
| 28 | 116.69 | 30.28 | Wuchang | 2012-2019 |
| 29 | 120.77 | 31.43 | Yangcheng | 2012-2019 |

# Table S6 Lakes with recorded TSI collected from published papers for validation.

| ID | LON. | LAT. | Lake name | Year | Reference |
| --- | --- | --- | --- | --- | --- |
| 1 | 88.96 | 44.88 | Aibi | 2010 | (Xin et al. 2010) |
| 2 | 119.14 | 33.27 | Baima | 1993, 2001-2003, 2005-2009 | (Chen et al. 2014) |
| 3 | 117.70 | 47.81 | Beier | 2011-2012 | (Hua 2014) |
| 4 | 87.04 | 41.97 | Bositeng | 1996-2006, 2009-2010 | (Song 2015) |
| 5 | 117.07 | 30.80 | Caizi | 2018 | (Zhu et al. 2019) |
| 6 | 124.26 | 45.27 | Chagan | 2004, 2007 | (Dong et al. 2007; Duan et al. 2006) |
| 7 | 87.90 | 43.50 | Chaiwobao | 2005-2009 | (Zhang 2011) |
| 8 | 117.53 | 31.57 | Chaohu | 2018 | (Zhu et al. 2019) |
| 9 | 100.66 | 26.55 | Chenghai | 1986-1999, 2006-2015 | (Chen et al. 2014) |
| 10 | 113.62 | 30.04 | Datong | 2018 | (Zhu et al. 2019) |
| 11 | 112.68 | 40.57 | Daihai | 1989, 1996, 2013 | (Sun 2008; Zhao et al. 2000 ) |
| 12 | 102.69 | 24.82 | Dianchi | 1988-2000, 2008-2010, 2019-2020 | (Chen et al. 2014) |
| 13 | 120.96 | 31.12 | Dianshan | 2018 | (Zhu et al. 2019) |
| 14 | 112.74 | 29.07 | Dongting | 2018 | (Zhu et al. 2019) |
| 15 | 100.18 | 25.82 | Erhai | 1992-2015, 2019-2020 | (Chen et al. 2014) |
| 16 | 102.89 | 24.52 | Fuxian | 1985-2000, 2004-2005 | (Li et al. 2020) |
| 17 | 119.29 | 32.85 | Gaoyou | 1993, 2008 | (Chen et al. 2014) |
| 18 | 119.81 | 31.60 | Gehu | 2018 | (Zhu et al. 2019) |
| 19 | 118.92 | 31.28 | Gucheng | 1987, 1993, 2001-2003, 2005, 2010 | (Chen et al. 2014) |
| 20 | 113.34 | 29.86 | Honghu | 2007-2016, 2018 | (Dong et al. 2016; Zhou 2017) |
| 21 | 118.59 | 33.31 | Hongze | 1991-1993, 1995-2000 | (Chen et al. 2014) |
| 22 | 117.40 | 48.94 | Hulun | 1987, 2003-2009, 2015-2017 | (Yang et al. 2019; Chen et al. 2014) |
| 23 | 116.38 | 30.02 | Huangda | 2018 | (Zhu et al. 2019) |
| 24 | 113.55 | 29.70 | Huanggai | 2018 | (Zhu et al. 2019) |
| 25 | 128.92 | 43.89 | Jingpo | 1988, 1991, 2000-2010 | (Chen et al. 2014) |
| 26 | 116.34 | 28.53 | Junshan | 2018 | (Zhu et al. 2019) |
| 27 | 114.51 | 30.23 | Liangzi | 2015 | (Dong et al. 2016; Zhu et al. 2019) |
| 28 | 116.15 | 29.95 | Longgan | 2018 | (Zhu et al. 2019) |
| 29 | 100.79 | 27.71 | Lugu | 2000 | (Chen et al. 2014) |
| 30 | 118.19 | 34.11 | Luoma | 1993, 1997-2005 | (Chen et al. 2014) |
| 31 | 113.12 | 29.34 | Nanhu | 2008-2009 | (Peng 2011) |
| 32 | 116.28 | 29.11 | Poyang | 1989, 1999, 2000, 2005 | (Huang 2010) |
| 33 | 102.77 | 24.17 | Qilu | 1991-2015 | (Li et al. 2020; Chen et al. 2014) |
| 34 | 81.17 | 44.60 | Sailimu | 2015 | (Luan 2017) |
| 35 | 119.43 | 32.62 | Shaobo | 1993, 2010 | (Chen et al. 2014) |
| 36 | 112.50 | 29.64 | Shengjin | 2018 | (Zhu et al. 2019) |
| 37 | 118.88 | 31.47 | Shijiu | 1995, 2001, 2002, 2010, 2018 | (Zhu et al. 2019; Chen et al. 2014) |
| 38 | 120.19 | 31.20 | Taihu | 1988, 1992-1995, 2006, 2018 | (Zhu et al. 2019; Chen et al. 2014) |
| 39 | 88.15 | 43.89 | Tianchi | 1993, 2010 | (Chen et al. 2014) |
| 40 | 108.85 | 40.96 | Wuliangsu | 2004-2007, 2011, 2013 | (Fan 2018) |
| 41 | 87.29 | 47.26 | Wulungu | 1987 | (Chen et al. 2014) |
| 42 | 116.69 | 30.28 | Wuchang | 2018 | (Zhu et al. 2019) |
| 43 | 115.59 | 29.91 | Wushan | 2018 | (Zhu et al. 2019) |
| 44 | 132.57 | 45.34 | Xiaoxingkai | 2012-2013 | (Yu et al. 2016) |
| 45 | 106.41 | 38.98 | Xinghai | 2011-2017 | (Ma et al. 2018) |
| 46 | 102.78 | 24.34 | Xingyun | 2000, 2019-2020 | (Li et al. 2020; Chen et al. 2014) |
| 47 | 132.29 | 45.13 | Xingkai | 1994-1998, 2001, 2007, 2009-2010 | (Liu et al. 2013; Piao and Wang. 2011) |
| 48 | 120.77 | 31.43 | Yangcheng | 2018 | (Zhu et al. 2019) |
| 49 | 103.00 | 24.91 | Yangzong | 2000 | (Chen et al. 2014) |
| 50 | 102.57 | 23.67 | Yilong | 2000 | (Chen et al. 2014) |
| 51 | 112.40 | 30.44 | Changhu | 2018 | (Zhu et al. 2019) |
| 52 | 116.67 | 29.14 | Zhuhu | 2018 | (Zhu et al. 2019) |

**References:**

Chen, X., Chuai, X., Yang, L. (2014). Status Quo, Historical Evolution and Causes of Eutrophication in Lakes in Typical Lake Regions of China. *Journal of Ecology and Rural Environment, 30*, 438-443

Duan, H.T., Yu, L., Zhang, B., Liu, D., Song, K., Wang, Z. (2006). Hyperspectral Data Applied in Monitoring and Evaluating the Water Trophic State of Lake Chagan. *Acta Sceientiae Circum Stantiae, 26*, 1219-1226.

Dong, J.W., Zhang, X.H. (2007). The Features and Development Trend of the Water Quality and Eutrophication of Cagan Lake. *Jilin Water Resources. 10*, 3-7.

Dong, W., M, S., Tang, L., Zeng, Q., Yang, J., Yao, M. (2016). Research of Eutrophication of Honghu Lake and Liangzi Lake. *Environmental Protection Science, 42*, 66-70

Fan, Y.L., Xing; Chun, Xi (2018). Research Progress of Eutrophication in Wuliangsuhai Sea. *Environmental Protection Science*, *44*, 83-88

Hua, L. (2014). Analysis of the Current Situation of Surface Water Environmental Quality in Hulun-Beier City. *Inner Mongolia Environmental Sciences, 26*, 191-193.

Huang, G.J. (2012). Remote Sensing Inversion Of Water Quality Parameters And Trophic State Assessment In Poynag Lake. In: Nanchang University

Li, Z., Chen, J., Yi, Q., Zhao, X., Lyu, Y., Zheng, X., Zhang, F., Hu, Z. (2020). Evolution and Change-Point Analysis of Trophic Status of the Fuxian Lake，the Xingyun Lake and the Qilu Lake. *Environmental Monitoring in China, 36*, 105-113

Liu, X.C., Li, X.P., Wang, Y.F., Wei, Z.M., Cheng, X., Chen, X.H. (2013). Simulation Of The Response Of Eutrophic State To Nutrient Input In Lake Xingkai Based On Ecological Dynamic Model. *Journal of Lake Sciences, 25*, 862-871.

Luan, H. (2017). Temporal And Spatial Distribution of Main Physical And Chemical Factors And Water Quality Assessment of Sayram Lake. In: Dalian Ocean University

Ma Z.P. (2018). Investigation and Cause Analysis of Eutrophication in Xinghai Lake. *Acta Agriculturae Shanghai,* 6, 18-22

Peng, X. (2011). Migration and transformation nutrient salts in north China lakes. In: Jilin University

Piao D.L., Wang, F.K. (2011). Environmental Conditions and the Protection Counter Measures for Waters of Lake Xingkai. *Journal of Lake Sciences, 23*, 196-202.

Song, Y. (2015). Analysis of water quality change trend in Bosten Lake. *Water Conservancy Science and Technology and Economy, 21*, 16-18

Sun, Y. (2008). Research of Endogenesis Release of Nitrogen And Phosphorous And Eutrophication In The Daihai Lake. In: Inner mongolia university

Xin, H.Y., Chen, Z.J., Wu, L. (2010). Evaluation of Water Quality and Nutrition Status of Ebinur Lake. *Journal of Salt Lake Research. 18*, 30-34,

Yang, Z., Li, C., Shi, X., Sun, B., Du, D., Quan, D. (2019). Study on the characteristics of water nutrition status and its main influencing factors in Hulun Lake. *Ecology and Environmental Sciences, 28*, 2273-2280

Yu, S.L., Li, X.J., Li, X.Y., Wen, B.L. (2016). Evaluation of Water Quality of Xiaoxinghai Lake. *Wetland Sceince, 11*, 466-469.

Zhang, Y. (2011). Analysis onTrophic Status and RestrictionFactors and Its Countermeasures Against Eutrophicationin Chai Wo-pu Lake. *Arid Environmental Monitoring, 25*, 160-166

Zhao, B., Cai, Q., Li, D., Liu, R. (2000). Preliminary Study on The Changes of Trophic State During The Process of Water Salinization In Daihai Lake, China. *ACTA HYDROBIOLOGICA SINICA*, 509-513

Zhou, Z. (2017). Study on Remote Sensing Retrieval of Honghu Lake in Recent ten years Based on GEE In: Hubei University

Zhu, G., Xu, H., Zhu, M., Zou, W., Guo, C., Ji, P., Da, W., Zhou, Y., Zhang, Y., Qin, B. (2019). Changing characteristics and driving factors of trophic state of lakes in the middle and lower reaches of Yangtze River in the past 30 years. *Journal of Lake Sciences, 31*, 1510-1524

# Supplementary Text 1

*A short review on the water quality in China’s lakes*

95% of the 19 surveyed lakes in the EPL were eutrophic and 37% were hypertrophic (Table S1). The average Chla in the EPL was 34.72 ± 49.61μg L^-1^. The correlation coefficient between SPIM and SPM was 0.94 (*p* < 0.01), and the concentration of SPIM accounted for more than 50% of that of SPM (N = 481). The SDD of 90% of the sampled lakes (N = 534) was lower than 0.5 m, showing typical characteristics of high turbidity eutrophication lakes in the EPL. In the YGPL, 60% of the 5 lakes surveyed were hyper eutrophic. The correlation between SPM and SPOM was elevated (R^2^ = 0.98, *p* < 0.01, N = 47), and the proportion of organic matter was high (~ 80% in average). Furthermore, a significant correlation between Chla concentration and SPOM was found (R^2^ = 0.95, *p* < 0.01, N = 47). The SDD of 17% of the sampled lakes (N = 199) was lower than 0.5 m.

# Supplementary Text 2

*Selection of benchmark for assessing eutrophication*

The correlation between Chla and TP in sampled lakes in China showed the algal growth was mainly affected by nutrients, as Chla had a good correlation with TP (R^2^ = 0.51, *p* < 0.01, N = 60). However, the SDD of sampled lakes is mainly affected by SPIM (R^2^ = 0.76, *p* < 0.01, N = 101), and showed no significant correlation with Chla (*p* > 0.05). The correlation between TSI(SDD) and TSI(Chla) were 0.50 (*p* < 0.01, N = 245, Fig. 3a). The relevance was improved as the joint of Chla (R^2^ = 86, *p* < 0.01, N = 245, Fig. 3b) and TP (R^2^ = 0.75, *p* < 0.01, N = 143, Fig. 3c). TSI(Chla, SDD, TP) showed the best correlation (R^2^ = 0.87, *p* < 0.01, N = 143, Fig. 3d) with the TSI(Chla) and the consistency of trophic level was 94.62%. Therefore, TSI derived from Chla does a good job of approximating the combination of SDD, Chla, and TP derived TSI.

**Fig. T2-1** Comparison of TSI based on Chla and (a) SDD, (b) SDD combing Chla, (c) SDD combing TP and (d) SDD combing Chla and TP

# Supplementary Text 3

*Classification of water pixel based on remote sensing reflectance*

Lakes were classified into algae dominated (Type 1), turbid (Type 2), and clean pixels (Type 3) to improve the precision of retrieved TSI model. Spectral curve characteristics of OLI surface reflectance for lakes with different trophic levels were compared. The remote sensing reflectance of high turbidity eutrophic lakes is relatively high, with the reflectance in the green band (561 nm) higher than that in the blue band (482 nm, Fig. T2-1a). The appearance of algal blooms significant increases in reflectance in NIR (865 nm, Fig. T2-1b). The spectral curve of oligotrophic lakes is significantly different from those of eutrophic and mesotrophic lakes (Fig. T2-1c, d). The spectral reflectance in the red band (665 nm) is reduced, nearing that in the NIR band (Fig. T2-1e, f). The reflectance in the blue band is higher than that in the green band, similar to that of clean marine water. In response to the spectral characteristics of these clean water pixels, 12 oligotrophic lakes were extracted and the differences in the blue, green band were compared. Among them, the reflectance in blue band of 10 lakes is greater than the green band, and the reflectance of visible light bands were lower than 0.10 defined by 95% confidence interval.

**Fig. T3-1** Spectral curve characteristics of OLI surface reflectance for lakes with different trophic levels: (a) Taihu Lake, (b) Hulun Lake, (c) Erhai Lake, (d) Wuliangsuhai, (e) Yangzhuoyong Co, (f) Bangong Co.
